# *ATP13A3* variants promote pulmonary arterial hypertension by disrupting polyamine transport

**DOI:** 10.1093/cvr/cvae068

**Published:** 2024-04-16

**Authors:** Bin Liu, Mujahid Azfar, Ekaterina Legchenko, James A West, Shaun Martin, Chris Van den Haute, Veerle Baekelandt, John Wharton, Luke Howard, Martin R Wilkins, Peter Vangheluwe, Nicholas W Morrell, Paul D Upton

**Affiliations:** Section of Cardio and Respiratory Medicine, Department of Medicine, Victor Phillip Dahdaleh Heart and Lung Research Institute, Papworth Road, Cambridge CB2 0BB, UK; Department of Cellular and Molecular Medicine, KU Leuven, Herestraat 49, Box 802, 3000 Leuven, Belgium; Section of Cardio and Respiratory Medicine, Department of Medicine, Victor Phillip Dahdaleh Heart and Lung Research Institute, Papworth Road, Cambridge CB2 0BB, UK; Cambridge Institute of Therapeutic Immunology and Infectious Disease, Jeffrey Cheah Biomedical Centre, Puddicombe Way, Cambridge CB2 0AW, UK; Division of Gastroenterology and Hepatology, Department of Medicine, Hills Road, Cambridge CB2 0QQ, UK; Department of Biochemistry and Cambridge Systems Biology Centre, University of Cambridge, Cambridge, UK; Department of Cellular and Molecular Medicine, KU Leuven, Herestraat 49, Box 802, 3000 Leuven, Belgium; Laboratory for Neurobiology and Gene Therapy, Department of Neurosciences, KU Leuven, Herestraat 49, Box 1023, 3000 Leuven, Belgium; Leuven Viral Vector Core, KU Leuven, Herestraat 49, Box 1023, 3000 Leuven, Belgium; Laboratory for Neurobiology and Gene Therapy, Department of Neurosciences, KU Leuven, Herestraat 49, Box 1023, 3000 Leuven, Belgium; Faculty of Medicine, National Heart and Lung Institute, ICTEM Building, Imperial College, Du Cane Road, London W12 0NN, UK; Faculty of Medicine, National Heart and Lung Institute, ICTEM Building, Imperial College, Du Cane Road, London W12 0NN, UK; Faculty of Medicine, National Heart and Lung Institute, ICTEM Building, Imperial College, Du Cane Road, London W12 0NN, UK; Department of Biochemistry and Cambridge Systems Biology Centre, University of Cambridge, Cambridge, UK; Section of Cardio and Respiratory Medicine, Department of Medicine, Victor Phillip Dahdaleh Heart and Lung Research Institute, Papworth Road, Cambridge CB2 0BB, UK; Section of Cardio and Respiratory Medicine, Department of Medicine, Victor Phillip Dahdaleh Heart and Lung Research Institute, Papworth Road, Cambridge CB2 0BB, UK

**Keywords:** ATP13A3, Pulmonary arterial hypertension, Polyamines

## Abstract

**Aims:**

Potential loss-of-function variants of *ATP13A3*, the gene encoding a P5B-type transport ATPase of undefined function, were recently identified in patients with pulmonary arterial hypertension (PAH). ATP13A3 is implicated in polyamine transport but its function has not been fully elucidated. In this study, we sought to determine the biological function of ATP13A3 in vascular endothelial cells (ECs) and how PAH-associated variants may contribute to disease pathogenesis.

**Methods and results:**

We studied the impact of *ATP13A3* deficiency and overexpression in EC models [human pulmonary ECs, blood outgrowth ECs (BOECs), and human microvascular EC 1], including a PAH patient–derived BOEC line harbouring an *ATP13A3* variant (LK726X). We also generated mice harbouring an *Atp13a3* variant analogous to a human disease–associated variant to establish whether these mice develop PAH. ATP13A3 localized to the recycling endosomes of human ECs. Knockdown of ATP13A3 in ECs generally reduced the basal polyamine content and altered the expression of enzymes involved in polyamine metabolism. Conversely, overexpression of wild-type ATP13A3 increased polyamine uptake. Functionally, loss of ATP13A3 was associated with reduced EC proliferation, increased apoptosis in serum starvation, and increased monolayer permeability to thrombin. The assessment of five PAH-associated missense ATP13A3 variants (L675V, M850I, V855M, R858H, and L956P) confirmed loss-of-function phenotypes represented by impaired polyamine transport and dysregulated EC function. Furthermore, mice carrying a heterozygous germline *Atp13a3* frameshift variant representing a human variant spontaneously developed a PAH phenotype, with increased pulmonary pressures, right ventricular remodelling, and muscularization of pulmonary vessels.

**Conclusion:**

We identify ATP13A3 as a polyamine transporter controlling polyamine homeostasis in ECs, a deficiency of which leads to EC dysfunction and predisposes to PAH. This suggests a need for targeted therapies to alleviate the imbalances in polyamine homeostasis and EC dysfunction in PAH.

## Graphical Abstract

**Graphical Abstract:**
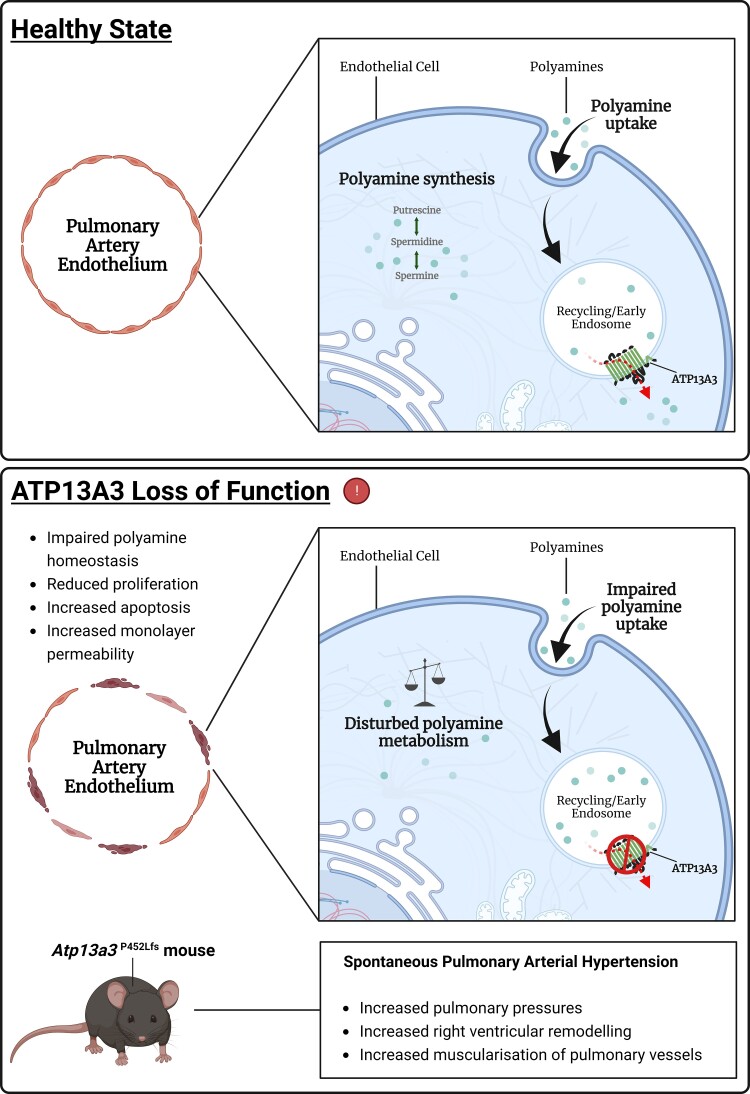

**Time for primary review: 39 days**

## 1. Introduction

Pulmonary arterial hypertension (PAH) is a progressive vascular disorder characterized by the narrowing and obliteration of small pre-capillary lung arterioles.^1^ Endothelial cell (EC) dysfunction, proliferation of mesenchymal cells in the vascular wall, and aberrant inflammation^1,2^ contribute to this pathological process. Despite the availability of licensed therapies, the survival of a patient with PAH remains poor, necessitating new targeted treatments.

The identification of heterozygous germline mutations in the bone morphogenetic protein Type II receptor (*BMPR2*) gene^3,4^ and the more recent identification of loss-of-function mutations in other BMP pathway components^5^ have underpinned potential PAH therapies to enhance BMP signalling.^6^ However, some rare PAH-related genes appear distinct from the BMP pathway,^5,7^ suggesting additional mechanisms underlying the pathobiology of PAH that may be informative for alternative therapies.

In a European-wide PAH cohort study, we identified 11 rare heterozygous *ATP13A3* variants with protein-truncating variants overrepresented (6 of 11), suggesting a loss of function in PAH.^7^ Since then, more *ATP13A3* variants have been reported in other PAH patient cohorts.^8–10^ Although *ATP13A3* is expressed in various cell types, including pulmonary vascular cells, pulmonary macrophages, and dendritic cells,^7,11,12^ its function remains unclear. *ATP13A3* is a member of the P5B-type ATPase family (ATP13A2-5), and ATP13A2 has recently been identified as a polyamine transporter.^13^ ATP13A3 has close homology to ATP13A2. Furthermore, recent studies have shown that *ATP13A3* mutations account for the polyamine uptake deficiency in CHO-MG cells,^14^ and ATP13A3 facilitates polyamine transport in human pancreatic cancer cells,^15^ strongly implicating ATP13A3 as a polyamine transporter.

Cellular polyamine levels are tightly regulated through the integration of their biosynthesis/catabolism and their transport, and disruption of these pathways can lead to diseases.^16,17^ In this study, we established that ATP13A3 mediates cellular polyamine uptake in human vascular ECs, whereas PAH-associated *ATP13A3* variants reduce polyamine transport. Loss of *ATP13A3* leads to PAH-associated phenotypes in pulmonary arterial ECs and in mice, harbouring a PAH-associated *Atp13a3* frameshift variant (P452Lfs). Collectively, our data explain the impact of *ATP13A3* variants in PAH and suggest that dysregulated polyamine homeostasis may contribute to its pathobiology.

## 2. Methods

Key protocols are described here, and additional detailed protocols are described in the Supplementary Material.

### 2.1 Animals

All animal procedures were performed in accordance with the Home Office Animals (Scientific Procedures) Act (1986) and were approved under Home Office Project Licence 70/8850. All studies were approved locally and conformed to the guidelines from Directive 2010/63/EU of the European Parliament on the protection of animals used for scientific purposes. The *Atp13a3* genetically modified mouse, C57BL/6Ntac-Atp13a3^em2H^/H (MGI: 6450011), designated *Atp13a3*^P452Lfs^, was generated at MRC Harwell using CRISPR-Cas9 editing to introduce a 1-nt deletion, resulting in a frameshift and termination codon after seven more amino acids (P452LfsTer7), equivalent to the human PAH variant P456Lfs.^7^

### 2.2 Haemodynamic assessments of mice

Cardiac catheterization was performed by the closed-chest technique, as described previously.^18^ Measurements of right ventricular systolic pressure (RVSP) were performed under isoflurane anaesthesia (2.0–2.5% isoflurane, 100% oxygen 2 L/min) in spontaneously breathing animals. In the same animals, systolic blood pressure in the aorta was measured. Mice were then killed by exsanguination while still under anaesthesia.

Hearts were excised, and the right ventricle (RV) was dissected, weighed, and then fixed in 10% neutral-buffered formalin. The lungs were inflated with 10% neutral-buffered formalin and harvested for histological analyses as described in the Supplementary Material.

Echocardiographic ultrasound measurements of heart rate, RV dimensions, and PA pressure surrogates were conducted in spontaneously breathing 6-month-old male mice under isoflurane anaesthesia using an ultrasound machine (Vevo 3100 System; FUJIFILM VisualSonics, Amsterdam, The Netherlands) equipped with a 40 MHz linear array transducer.

### 2.3 Cell culture

Human pulmonary artery ECs (hPAECs) were purchased from Promocell (Heidelberg, Germany) and maintained in Endothelial Growth Medium (EGM)2 media (Promocell) with 2% foetal bovine serum (FBS) and antibiotics/antimycotics, in accordance with the supplier’s instructions.

Human blood outgrowth ECs (BOECs) were isolated from 40 to 80 mL of blood, as previously described.^19^ BOEC lines were grown in EGM2 with the addition of 10% FBS, antibiotics/antimycotics, and omission of heparin. For experiments involving BOEC generation, all donors provided informed written consent in accordance with the Declaration of Helsinki under human study 07/H0306/134 (Cambridgeshire 3 Research Ethics Committee) or REC—17/LO/0563 (*ATP13A3*-LK726X variant carrier). Demographic and variant information for the BOECs used in this study is specified in Supplementary material online, *Table S1*, and genomic information for the LK726X variant is detailed in Supplementary material online, *Table S2*. Both hPAECs and BOECs were used for experiments between Passages 4 and 7.

The immortalized (SV40-transformed) human microvascular EC-1 (HMEC-1) line was purchased from ATCC (Manassas, VA). HMEC-1 were grown in MCDB131 medium (without glutamine; Thermo Fisher Scientific, Waltham, MA) supplemented with 1 µg/mL Hydrocortisone (Sigma-Aldrich, St Louis, MO), 10 mM Glutamine (Sigma-Aldrich), 10 ng/mL Epidermal Growth Factor (R&D Systems, Minneapolis, MN), and 10% (v/v) FBS and antibiotics/antimycotics. All cells were routinely tested for mycoplasma and were used only if they were negative.

### 2.4 Cellular transfection and transduction

Cells were transfected with *ATP13A3* siRNA and *ATP13A3* expression plasmids or transduced with lentiviral expression particles,^20^ as described in the Supplementary Material. All variants studied are detailed in Supplementary material online, *Table S2*.

### 2.5 Measurement of cellular polyamines

Aqueous metabolites were extracted from cell lysates and analysed for polyamine content by liquid chromatography–mass spectrometry (LC–MS), as described in the Supplementary Material.

### 2.6 BODIPY-labelled polyamine uptake assay

BODIPY-tagged spermine (SPM-BDP), spermidine (SPD-BDP), and putrescine (PUT-BDP) were synthesized, as previously described,^21^ and dissolved in 0.1 M 3-morpholinopropane-1-sulfonic acid (MOPS), pH = 7.0 (AppliChem, A1076, Darmstadt, Germany). The uptake of polyamine-BDP in HMEC-1 was determined by flow cytometry. HMEC-1 cells were seeded in 12-well plates at 300 000 cells/well and left to attach overnight. After determining the kinetics of uptake to ensure that the cells were in the linear phase, they were incubated with the respective polyamine-BDP concentration (5 µM, if it was a single concentration) for 30 min after which they were trypsinized, and centrifuged at 300*×g*, and the pellet was washed with cold Dulbecco’s phosphate-buffered saline (PBS) solution without calcium or magnesium (Sigma, D8537). The pellets were then re-suspended in 1% bovine serum albumin/PBS. Polyamine-BDP uptake was determined by flow cytometry on a BD FACSCanto™ II instrument (BD Life Sciences, Franklin Lakes, NJ), with 10 000 events recorded per treatment.

### 2.7 Statistical analysis

The data are presented as mean ± standard error of the mean (SEM) and are analysed using GraphPad Prism 7 (GraphPad Software, Boston, MA). All presented data are *n* = 3 (unless mentioned otherwise), where *n* represents the number of independent repeats. The Kolmogorov–Smirnov test was performed for assessing normality, and the data were analysed by one-way analysis of variance (ANOVA) with *post hoc* Tukey’s honestly significant difference (HSD) analysis, one-/two-way ANOVA followed by multiple comparisons using Dunnett’s/Tukey’s *post hoc* tests, or an unpaired two-tailed Student’s *t*-test as indicated. A value of *P* < 0.05 was considered statistically significant.

## 3. Results

### 3.1 ATP13A3 is a polyamine transporter localized to recycling endosomes

**Figure 1:**
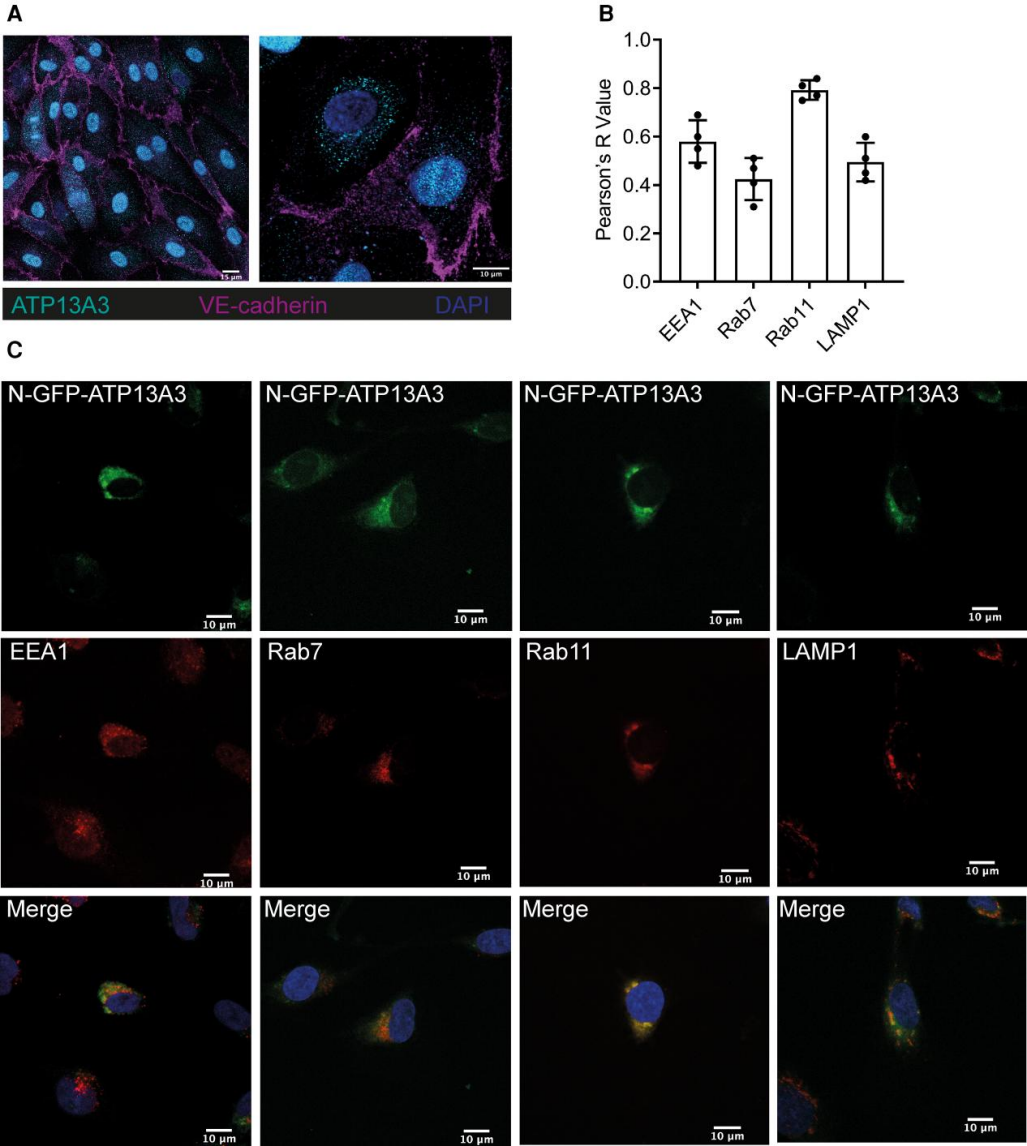
ATP13A3 is a polyamine transporter residing in the recycling endosome of ECs. (*A*) Confocal images at ×40 (left panel, scale bar = 15 µm) and ×63 (right panel, scale bar = 10 µm) of hPAECs costained with anti-ATP13A3 and anti-VE-Cadherin. (*B*) Pearson’s coefficients of the correlation of GFP-tagged ATP13A3 to different endosomal markers in HMEC-1 cells. (*C*) Confocal images (×63, scale bar = 10 µm) of HMEC-1 cells transiently overexpressing hATP13A3-N-GFP-pcDNA6.2 costained with antibodies against EEA1, Rab7, Rab11, or LAMP1. The data are representative of *n* = 4 experiments.

**Figure 2:**
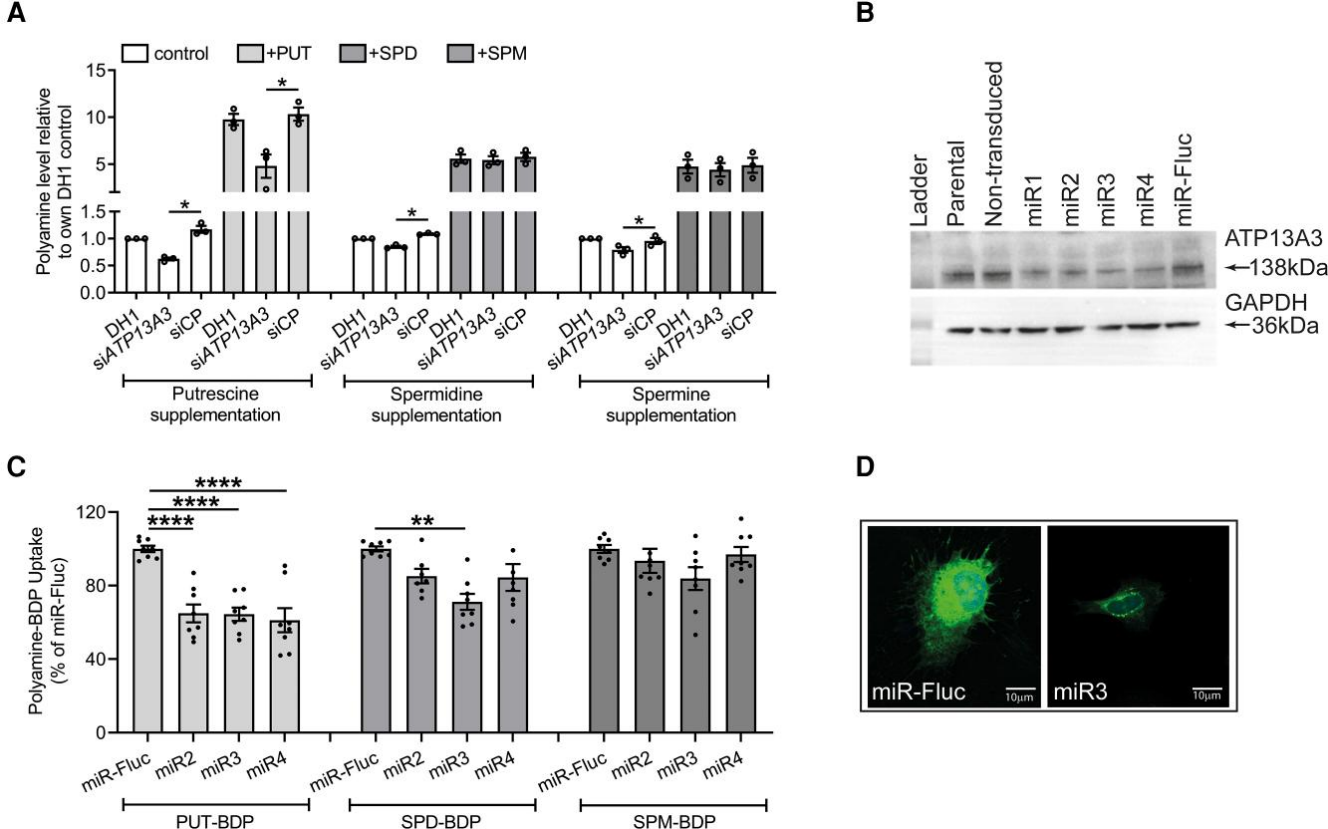
*ATP13A3* deficiency impairs polyamine transport in ECs. (*A*) Cellular PUT, SPD, and SPM levels in hPAECs measured by LC–MS. Cells were transfected with DharmaFECT 1™ (DH1, Cambridge, UK) alone, si*ATP13A3*, or non-targeting siRNA control (siCP) and cultured overnight in EBM2 containing 2% FBS supplemented with or without 1 mM PUT, 10 µM, SPD, or 10 µM SPM. The data (*n* = 3 experiments) are presented as polyamine peak area ratio relative to 2% FBS DH1. (*B*) Western blot showing ATP13A3 protein expression in parental, non-transduced, and HMEC-1 cells stably expressing miRNAs targeting *ATP13A3* (miR1–miR4), with miR-FLUC (Firefly Luciferase) as a control. (*C*) BDP-labelled polyamine uptake in HMEC-1 stable knockdown lines (*n* = 4 experiments, two technical replicates per experiment). The data are normalized to the mean fluorescent intensities of miR-FLUC. (*D*) Confocal microscopy depicting the uptake and distribution of PUT-BDP in HMEC-1 cells, expressing miR-FLUC and *ATP13A3* miR3 following 2 h treatment with PUT-BDP (scale bar = 10 µm). (*A*, *C*) The data (mean ± SEM) were analysed using a one-way ANOVA with Tukey’s *post hoc* test for multiple comparisons. *P < 0.05, **P < 0.01, ****P < 0.0001.

P5B-ATPases are multi-span transmembrane proteins that may localize to the plasma membrane^15^ or endosomal system.^22^ Endogenous ATP13A3 in hPAECs localized primarily to a perinuclear region with lower nuclear staining (*Figure 1A*). Both Green fluorescent protein (GFP)-tagged (*Figure 1B* and *C*) and endogenous (see Supplementary material online, *Figure S1*) ATP13A3 in HMEC-1 cells colocalized mainly with Rab11, a Ras-like GTPase critically important in vesicle trafficking, although some colocalizations were also observed with EAA, LAMP1, and Rab7 (*Figure 1B* and *C* and Supplementary material online, *Figure S1*). These data suggest ATP13A3 shows a general expression pattern in the endolysosomal system, with the highest colocalization observed in recycling endosomes (Rab11).We hypothesized that ATP13A3 mediates polyamine transport in primary human ECs. *ATP13A3* siRNA (si*ATP13A3*) in hPAECs reduced *ATP13A3* expression without affecting the expression of *ATP13A1-2* (see Supplementary material online, *Figure S2A–C*). We could not detect *ATP13A4-5* expression in these cells. si*ATP13A3* significantly reduced the basal cellular PUT, SPM, and SPD contents in hPAECs, while only the increase in PUT content was significantly attenuated upon exogenous polyamine supplementation (*Figure 2A*). For validation, we stably silenced *ATP13A3* in HMEC-1 cells (HMEC-1^mi*ATP13A3*^) using three lentiviral micro-RNAs (miRNA), targeting different regions of the *ATP13A3* mRNA (*Figure 2B* and Supplementary material online, *Figure S2D* and *E*). Like in hPAECs, basal PUT, SPD, and SPM contents were reduced in HMEC-1^mi*ATP13A3*^ cells (see Supplementary material online, *Figure S2F*) and uptake of PUT-BDP and SPD-BDP, but not SPM-BDP was impaired (*Figure 2C*). Furthermore, confocal imaging confirmed lower PUT-BDP uptake in HMEC-1^mi*ATP13A3*^, with the internalized polyamine predominantly confined to punctae (*Figure 2D*). In conclusion, ATP13A3 determines polyamine uptake, redistribution, and homeostasis in ECs.

### 3.2 ATP13A3 levels alter the expression of polyamine biosynthesis pathways

**Figure 3:**
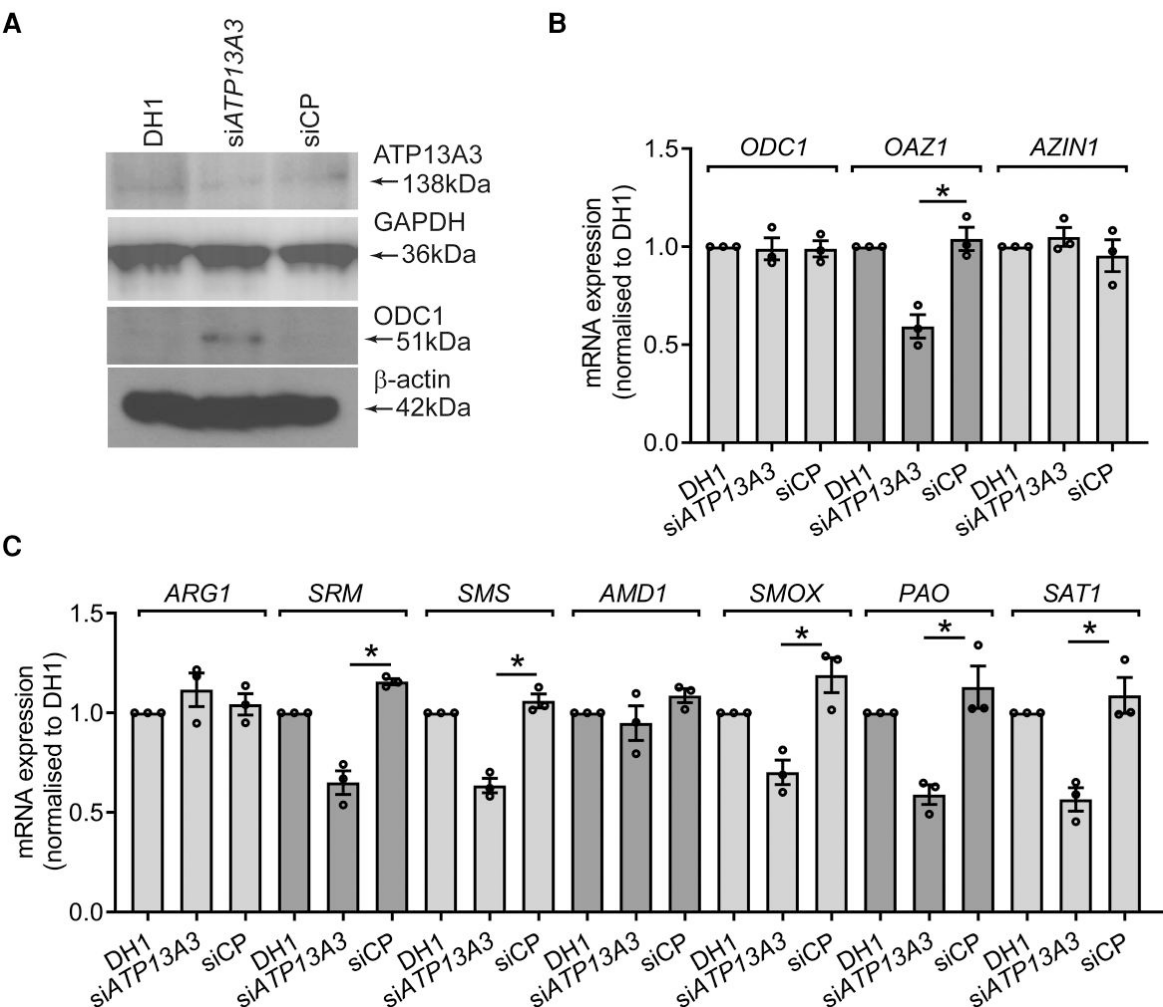
*ATP13A3* deficiency affects polyamine metabolism in hPAECs. (*A*) Immunoblotting for ATP13A3 and ODC1 in hPAECs after transfection with DH1 alone, si*ATP13A3*, or non-targeting siCP. (*B*, *C*) Transcription of (*B*) *ODC1*, *OAZ1*, *AZIN1*, and polyamine biosynthesis enzymes (*ARG1*, *SRM*, *SMS*, and *AMD1*) and (*C*) catabolic enzymes (*SMOX*, *PAO*, and *SAT1*) in hPAECs transfected with DH1, si*ATP13A3*, or siCP. The data (*n* = 4 experiments) are fold-change relative to the DH1 control for each transcript. (*B*, *C*) The data (mean ± SEM) were analysed using a one-way ANOVA with Tukey’s *post hoc* test for multiple comparisons. *P < 0.05 compared with siCP.

Polyamine homeostasis is maintained through the integrated functions of polyamine transporters and polyamine metabolism enzymes (see Supplementary material online, *Figure S3*). Ornithine decarboxylase (ODC) converts ornithine into PUT to initiate polyamine biosynthesis and is tightly regulated by cellular polyamine levels.^17^ ODC protein levels increased without altering mRNA expression in si*ATP13A3-*transfected hPAECs or BOECs (*Figure 3A* and *B* and Supplementary material online, *Figures S4* and *S5A–C*). Moreover, expression of antizyme (*OAZ1*), which mediates the polyamine-dependent proteasomal degradation of ODC,^23^ was reduced, whereas the antizyme inhibitor (AZIN) was unchanged (*Figure 3B* and Supplementary material online, *Figure S5C*). Hence, the increased ODC protein in *ATP13A3* deficiency may occur via *OAZ1* down-regulation.

In si*ATP13A3*-transfected hPAECs and BOECs, arginase 1 (*ARG1*) and adenosylmethionine decarboxylase (*AMD1)* expression were unchanged, whereas SPD synthase (*SRM*) and SPM synthase (*SMS*) were reduced (*Figure 3C* and Supplementary material online, *Figure S5D*). Also, the expression of the polyamine catabolic enzymes, SPD/SPM N1-acetyltransferase 1 (*SAT1*), and polyamine oxidase (*PAO*) was lower in si*ATP13A3*-transfected cells, possibly as an attempt by the cells to rebalance PUT levels (*Figure 3C* and Supplementary material online, *Figure S5D*).

### 3.3 ATP13A3 deficiency leads to pulmonary artery endothelial dysfunction

**Figure 4:**
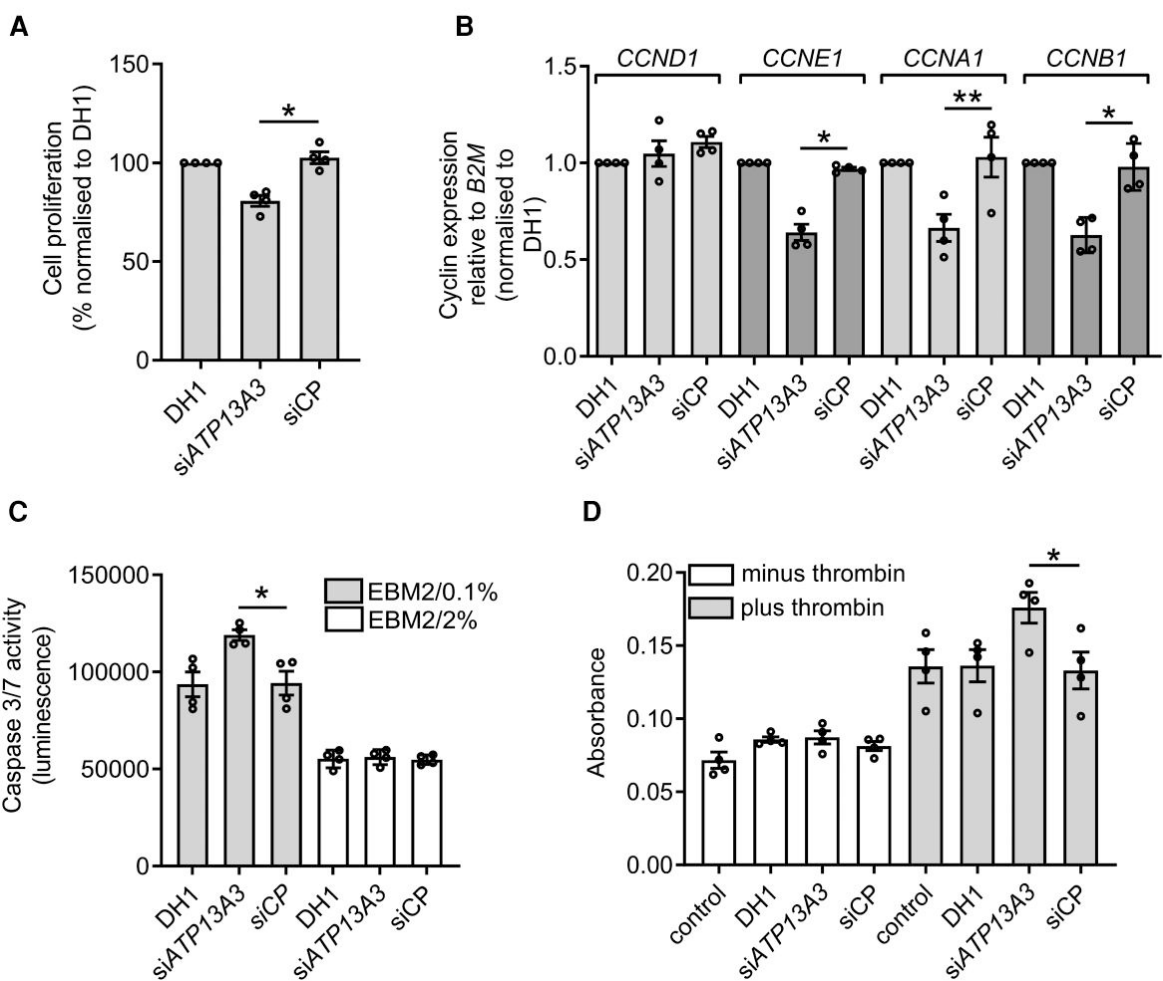
*ATP13A3* deficiency leads to endothelial dysfunction. (*A*) Proliferation, determined by cell counting, of transfected hPAECs over 6 days in EBM2 with 2% FBS, with media replenished every 2 days. (*B*) Transcription of *CCND*, *CCNE*, *CCNA*, and *CCNB* mRNAs with *ATP13A3* deficiency assessed by quantitative polymerase chain reaction. The data are fold-change relative to the DH1 control for each transcript. (*C*) Apoptosis assessed by Caspase-Glo®3/7 assay (Promega, Madison, NI) of transfected hPAECs cultured in EBM2 with 0.1% FBS or 2% FBS. (*D*) Permeability of transfected hPAEC monolayers to horseradish peroxidase in the absence or presence of 1 U/mL thrombin assessed by colorimetric assay. The data are the raw absorbance values for the different groups at the 2 h time point. The data (*n* = 4 experiments) in *A*–*D* are mean ± SEM and were analysed using a one-way ANOVA with Tukey’s *post hoc* test for multiple comparisons. *P < 0.05, **P < 0.01 compared with siCP.

Dysregulated proliferation and increased apoptosis and permeability of ECs contribute to the pathobiology of PAH.^1,2^ We previously reported that *ATP13A3* knockdown in BOECs impaired their proliferation and increased apoptosis in reduced serum.^7^ Here, we established by cell counting that si*ATP13A3* reduced hPAEC proliferation (*Figure 4A*), which was associated with reduced mRNA expression of cyclins E (*CCNE1*), A (*CCNA1*), and B (*CCNB1*), suggesting impaired cell cycle progression (*Figure 4B*). Supplementation with 10 µM PUT, SPD, or SPM promoted hPAEC proliferation (see Supplementary material online, *Figure S6A–C*), which was attenuated in si*ATP13A3*-transfected hPAECs (see Supplementary material online, *Figure S6D*). The reduced proliferation in Endothelial Basal Medium-2 (EBM)2/2% FBS was not due to increased apoptosis, as caspase 3/7 activity was not altered by *ATP13A3* knockdown (*Figure 4C*). However, caspase 3/7 activity was increased by si*ATP13A3* in hPAECs incubated in low serum conditions, suggesting a greater susceptibility to intrinsic stress (*Figure 4C*). Moreover, si*ATP13A3* did not alter basal hPAEC monolayer permeability, but we observed 40% higher permeability when monolayers were exposed to 1 U/mL thrombin (*Figure 4D*).

### 3.4 PAH-associated variants impair ATP13A3-mediated polyamine uptake in HMEC1 cells and hPAECs

To establish whether PAH-associated *ATP13A3* variants are pathogenic, we assessed the functional impact of five PAH-associated missense protein variants (L675V, M850I, V855M, R858H, and L956P; Supplementary material online, *Figure S7A*) in different EC models, comparing these with ATP13A3 wild-type (ATP13A3-WT) protein and an artificial transport dead mutant protein with a D498N substitution in the catalytic autophosphorylation domain.

**Figure 5:**
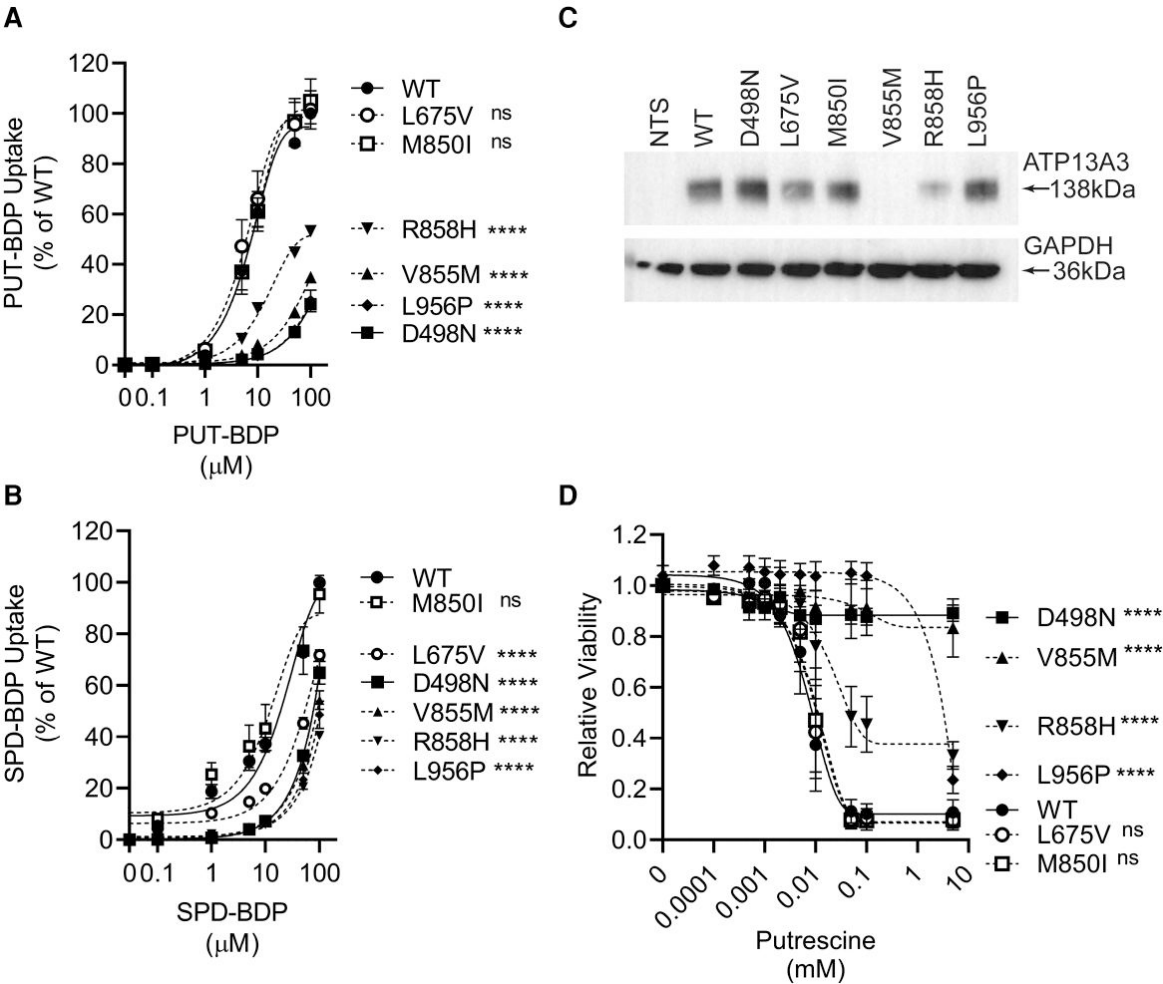
PAH-associated *ATP13A3* variants exhibit deficient polyamine uptake. (*A*, *B*) Flow-cytometric analysis for the assessment of cellular uptake of increasing concentrations of (*A*) PUT-BDP (*n* = 5 experiments) and (*B*) SPD-BDP (*n* = 6 experiments) after 30 min exposure. The data are normalized to WT. (*C*) Western blot showing ATP13A3 protein expression in non-transduced (NTS) HMEC-1 cells compared with those stably expressing untagged ATP13A3-WT (WT), an artificial transport dead mutant (D498N) or five PAH-associated variants (L675V, M850I, V855M, R858H, and L956P). (*D*) Cytotoxicity (MUH reagent) assay with a concentration–response analysis to PUT (*n* = 4 experiments). (*A*, *B*, *D*) The data were analysed by two-way ANOVA followed by multiple comparisons using Tukey’s *post hoc* tests. ****P < 0.00005 and ns = non-significant.

Stable lentiviral overexpression in HMEC1 cells of the untagged WT, but not the artificial D498N mutant protein, increased the uptake of PUT-BDP and SPD-BDP, but not SPM-BDP (*Figure 5A* and *B* and Supplementary material online, *Figure S7B*), consistent with our knockdown results (*Figure 2C*). Benzyl Viologen, a polyamine uptake inhibitor,^14^ blocked PUT-BDP uptake (see Supplementary material online, *Figure S7C*).

Interestingly, despite the mRNA expression of the V855M variant being comparable with the WT and the R858H being higher than the WT (see Supplementary material online, *Figure S7E*), both variants showed reduced protein expression (*Figure 5C* and Supplementary material online, *Figure S7F*). As the antibody epitope (488–631) lies outside the mutated region, this implies reduced protein stability. Consequently, these variants fail to increase PUT-BDP and SPD-BDP uptake *Figure 5A* and *B*). Though the other variant proteins expressed well, the uptake of both PUT-BDP and SPD-BDP was impaired only for the L956P variant protein. Intriguingly, the L675V variant protein only demonstrated reduced SPD-BDP uptake, suggesting altered substrate specificity.

Polyamines are essential for cell survival but at high concentrations, can be cytotoxic.^13^ Stable ATP13A3-WT protein overexpression sensitized HMEC-1 cells to escalating PUT concentrations, whereas the artificial D498N mutant did not (*Figure 5D*). Mirroring their impact on PUT-BDP uptake, the three untagged PAH-associated protein variants (V855M, R858H, and L956P) also exhibited reduced sensitivity to PUT toxicity.

So far, the M850I protein variant did not differ from the WT, although the endogenous PUT, SPD, and SPM levels were reduced in HMEC-1 cells stably overexpressing the M850I, L956P protein variants, and artificial D498N mutant, albeit non-significantly (see Supplementary material online, *Figure S8A–C*). However, stable overexpression of the L675V and M850I missense variant proteins did significantly decrease basal SPD and SPM levels in neuroblastoma SH-SY5Y cells (see Supplementary material online, *Figure S8D–F*), suggesting a cell-type-specific phenotype for these variants.To analyse the intracellular localization of the protein variants, we transiently overexpressed GFP-tagged WT, D498N, and the five PAH-associated ATP13A3 protein variants in HMEC-1 cells. Surprisingly, all GFP-tagged variants and WT-GFP consistently colocalized with Rab11, suggesting that when transiently expressed, V855M-GFP and R858H-GFP express well (see Supplementary material online, *Figure S9A*). In hPAECs transiently overexpressing these constructs, basal PUT levels were similar to all variants (see Supplementary material online, *Figure S9B*). Supplementation with 1 mM PUT increased endogenous PUT levels mainly in WT-GFP expressing cells, since the ATP13A3 protein variants attenuated (R858H-GFP and L956P-GFP) or abolished (D498N-GFP, L675V-GFP, M850I-GFP, and V855M-GFP) this response (see Supplementary material online, *Figure S10C*). We confirmed similar *ATP13A1-3* expression of all the constructs (see Supplementary material online, *Figure S10A–C*).

We further assessed the impact of high polyamine concentrations on apoptosis (caspase-3/7 activity) of hPAECs. Although ATP13A3-WT-GFP overexpression sensitized hPAECs to 10 mM PUT (see Supplementary material online, *Figure S11A*), this was reduced for the artificial D498N-GFP mutant and disease variants L675V-GFP, M850I-GFP, and V855M-GFP, consistent with the attenuated response to PUT supplementation in these cells. In contrast, the R858H-GFP and L956P-GFP variants behaved more akin to the ATP13A3-WT (see Supplementary material online, *Figure S11A*). No differences were seen among ATP13A3-WT, the D498N, and PAH-related protein variants to SPD and SPM toxicity (see Supplementary material online, *Figure S11B* and *C*).

Together, our analysis of complementary expression systems reveals that the ATP13A3 missense variants present different forms of loss-of-function phenotypes, affecting polyamine uptake and/or homeostasis.

### 3.5 The ATP13A3^LK726^ frameshift variant predisposes BOECs to apoptosis by affecting ATP13A3-mediated polyamine transport

**Figure 6:**
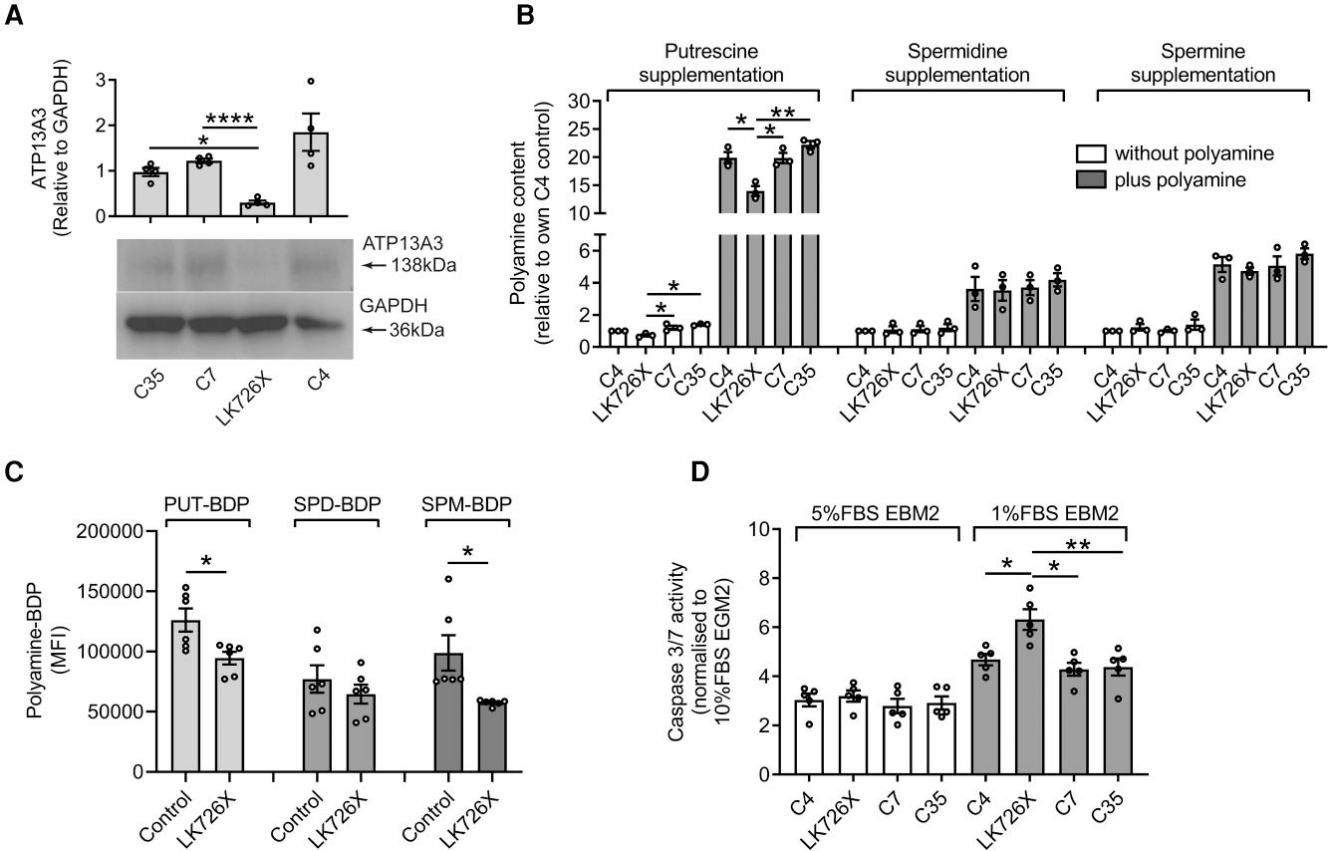
The *ATP13A3* LK726X frameshift variant predisposes BOECs to apoptosis by affecting ATP13A3-mediated polyamine transport. (*A*) Immunoblotting of ATP13A3 in control BOECs (C4, C7, C35) and *ATP13A3*^LK726X^ BOECs. Densitometric analysis of ATP13A3 and α-tubulin was performed (graph, *n* = 4 experiments). (*B*) Cellular polyamine contents, measured by LC–MS, of BOECs in media or supplemented with 1 mM PUT, 10 µM SPD, or 10 µM SPM. The data (*n* = 3 experiments) are presented as polyamine peak area ratio relative to the C4 control BOEC line. (*C*) BOEC uptake of PUT-BDP, SPD-BDP, and SPM-BDP measured by flow cytometry (*n* = 3 experiments, two technical replicates per experiment). (*D*) Cell apoptosis of BOECs cultured in EBM2 supplemented with 1% FBS or 5% FBS was assessed by Caspase-Glo®3/7 assay (*n* = 5 experiments). The data are normalized to cells cultured in EGM2 containing 10% FBS. (*A–D*) The data are mean ± SEM analysed using (*A*, *B*, *D*) one-way ANOVA with Tukey’s *post hoc* test for multiple comparisons or (*C*) unpaired *t*-test. *P < 0.05, **P < 0.01, ****P < 0.0001 compared with *ATP13A3*^LK726X^.

To cross-validate our findings in a disease-relevant endogenous system, we derived BOECs from a patient with PAH bearing a heterozygous *ATP13A3* frameshift variant (*ATP13A3*^LK726X^, c.2176_2180delTTAAA), confirmed by Sanger sequencing (see Supplementary material online, *Figure S12*). This variant creates a premature stop codon (TGA 733X), predicted to reduce *ATP13A3* mRNA expression through nonsense-mediated decay. In support of this, both *ATP13A3* mRNA and protein levels (*Figure 6A* and Supplementary material online, *Figure S13A*) were reduced in *ATP13A3*^LK726X^ BOECs compared with control BOECs, without changes in the expression of *ATP13A1* and *ATP13A2* (see Supplementary material online, *Figure S13B* and *C*). Compared to control cells, *ATP13A3*^LK726X^ BOECs exhibited lower PUT content under both basal and PUT-supplemented conditions, though the fold increase in PUT content with supplementation was similar for all the BOEC lines (*Figure 6B*). Although basal and supplemented SPM and SPM contents were unchanged (*Figure 6B*), the uptake of PUT-BDP and SPM-BDP was significantly lower than in control BOECs (*Figure 6C*). Functionally, caspase-3/7 activity in low serum was significantly elevated in *ATP13A3*^LK726X^ BOECs (*Figure 6D*), and this was partially rescued by overexpression of the ATP13A3-WT protein, but not the artificial D498N mutant (see Supplementary material online, *Figure S14*).

Interestingly, *ODC* mRNA and protein levels were increased in *ATP13A3*^LK726X^ BOECs (Supplementary material online, *Figure S15A* and *B*), which was not explained by changes in *OAZ1* or *AZIN1* expression (see Supplementary material online, *Figure S15C* and *D*). Intriguingly, the expression of the synthetic enzymes, *ARG1*, *AMD1*, *SRM*, and *SMS*, were also elevated (see Supplementary material online, *Figure S15E*), while the catabolic enzymes remained comparable between *ATP13A3*^LK726X^ and control BOECs (see Supplementary material online, *Figure S16*). Collectively, our data suggest the *ATP13A3*^LK726X^ variant disrupts polyamine homeostasis in BOECs, predisposing cells to apoptosis.

### 3.6 Mice harbouring an Atp13a3^P452Lfs^ variant spontaneously develop PAH

To explore the potential role of potentially pathogenic ATP13A3 variants in PAH, we generated a new mouse line harbouring an *Atp13a3* variant (P452LfsTer7), homologous to a human disease–associated *ATP13A3* variant (P456Lfs).^7^ The mice were viable and fertile, although homozygous males and females were born at a lower frequency than the expected Mendelian ratio [males (*n* = 110): 26.4% Wt: 57.3%, Het: 16.3% Hom; females (*n* = 107): 28.0% Wt: 56.1%, Het: 15.9% Hom]. Otherwise, mice harbouring heterozygous or homozygous *Atp13a3*^P452LfsTer7^ alleles did not exhibit any overt behavioural abnormalities, nor did they differ in appearance or weight from their WT littermates.

**Figure 7:**
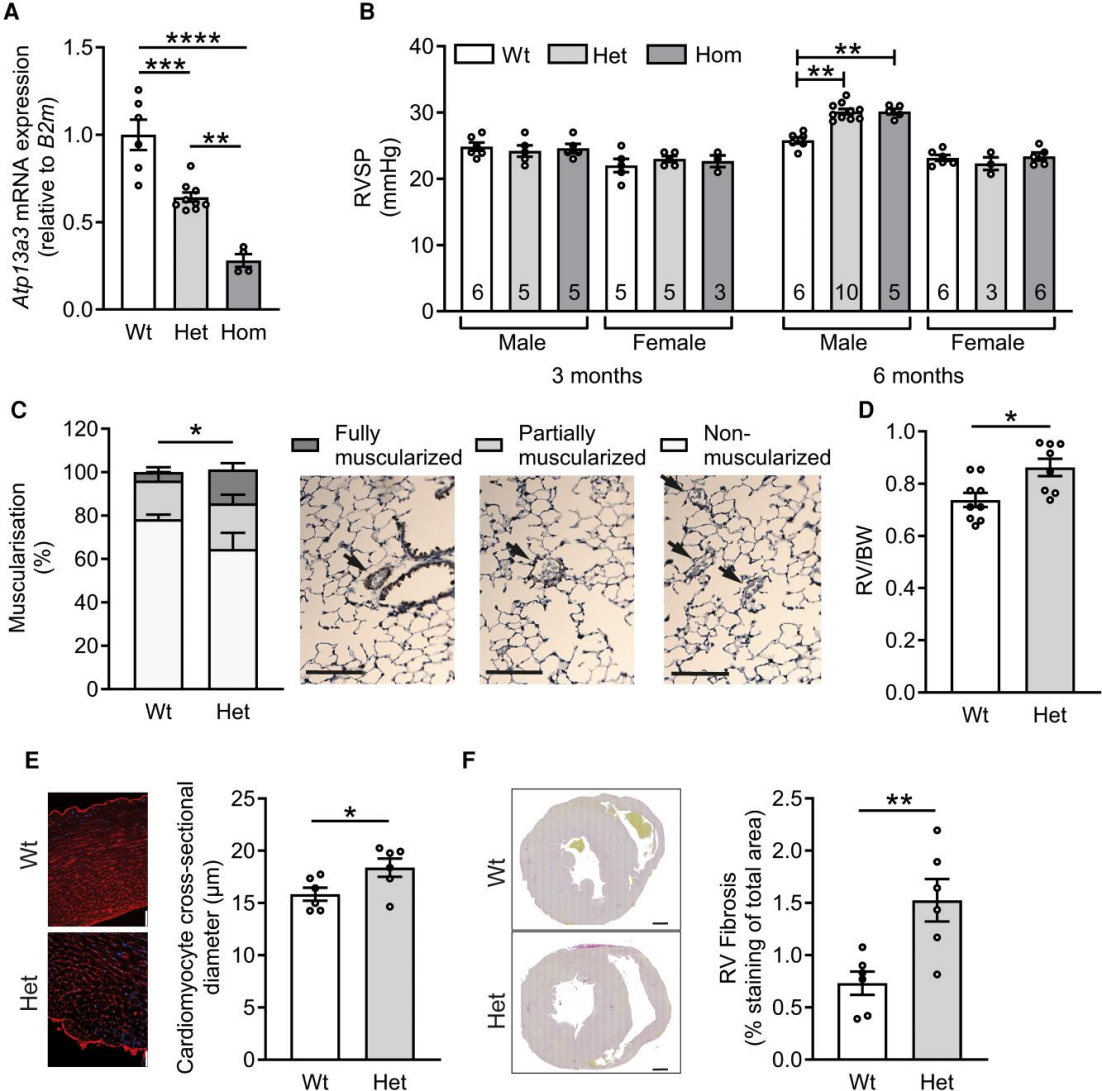
Male mice harbouring an *Atp13a3*^P452Lfs^ variant spontaneously develop PAH at 6 months of age. (*A*) Expression of *Atp13a3* mRNA in whole lungs of male *Atp13a3*^P452Lfs^ heterozygous (Het, *n* = 9) and homozygote (Hom, *n* = 4) mice compared with WT littermate controls (Wt, *n* = 6). (*B*) Invasive haemodynamic measurement of RVSP in male and female *Atp13a3*^P452Lfs^ heterozygous and homozygous mice and WT littermate controls aged 3 or 6 months (numbers in bars). (*C–F*) Further comparison of male heterozygous *Atp13a3*^P452Lfs^ mice and WT littermate controls aged 6 months. (*C*) The assessment of vessel muscularization of arterioles <50 μm in diameter (*n* = 4 Wt and 10 Het mice, *n* = 12–33 vessels/lung) as indicated by arrows in the respective α-smooth muscle actin immunostaining panels (scale bars = 100 µm). (*D*) Ratio of RV weight to bodyweight (BW) (*n* = 9, 8). (*E*) Wheat-germ agglutinin staining of cardiomyocytes in heart sections (scale bar = 50 µm) from which the cardiomyocyte cross-sectional areas were measured (graph, *n* = 6 per group). (*F*) RV fibrosis in picrosirius red-stained sections (panels show representative images, scale bar = 250 µm). Fibrosis was measured as the percentage of red staining in isolated RV images (11–20 images per heart, *n* = 6 animals per group). The data are mean ± SEM and were analysed with: (*A*) one-way ANOVA with Tukey’s multiple comparison test; (*B*) Kruskal–Wallis test with multiple comparisons or: (*C–F*) a two-tailed unpaired *t*-test with Welch’s correction. *P < 0.05, **P < 0.005, ***P < 0.0005, ****P < 0.0001.

Male mice heterozygous or homozygous for the *Atp13a3*^P452LfsTer7^ allele exhibited reduced lung *Atp13a3* expression reflecting their genotypes (*Figure 7A*). When RVSPs were assessed in male and female mice aged to 3 months, there were no genotype-related differences (*Figure 7B*). When aged to 6 months of age, female mice carrying either heterozygous or homozygous *Atp13a3*^P452LfsTer7^ exhibited identical pressures to WT littermate controls. However, heterozygous male *Atp13a3*^P452Lfs^ mice spontaneously and consistently developed pulmonary hypertension (PH) compared with their littermate controls at 6 months of age, with a significant elevation of RVSP (*Figure 7B*), while heart rate and systemic blood pressure did not differ (see Supplementary material online, *Figure S17A* and *B*). Transthoracic echocardiography demonstrated a significant shortening of pulmonary artery acceleration time (a surrogate of pulmonary artery pressure and pulmonary vascular resistance) in heterozygous *Atp13a3*^P452Lfs^ mice (see Supplementary material online, *Figure S17C*). Heterozygous *Atp13a3*^P452Lfs^ mice also exhibited increased right heart dimensions, namely RV inner diameter (see Supplementary material online, *Figure S17D*) and RV end-diastolic anterior wall thickness (see Supplementary material online, *Figure S17E*) compared with the WT littermates. Again, heart rate did not differ between the two groups (see Supplementary material online, *Figure S17F*).

Consistent with the haemodynamic data suggesting a baseline PAH phenotype, we also observed histological changes in the pulmonary circulation and hearts of *Atp13a3*^P452Lfs^ mice. Lung vascular morphometric analysis revealed that heterozygous *Atp13a3*^P452Lfs^ mice had a significantly higher percentage of fully muscularized small pulmonary arteries (*Figure 7C*). Consistent with this phenotype, the RVs were also hypertrophic (*Figure 7D*). This was associated with a significant increase in cardiomyocyte cross-sectional diameter (*Figure 7E*) and in interstitial fibrosis (*Figure 7F*). Although milder than the PAH observed in induced models of disease, these data confirmed that *Atp13a3*^P452Lfs^ heterozygous mice spontaneously and consistently demonstrated a PAH phenotype without the need for an applied disease-promoting stimulus.

## 4. Discussion

In this study, we establish ATP13A3 in ECs as an endolysosomal polyamine transporter enriched in recycling endosomes. ATP13A3 enables cellular polyamine uptake and ATP13A3 deficiency causes EC dysfunction. Human disease–associated *ATP13A3* variants present a loss of polyamine uptake phenotype, whereas male mice harbouring a human disease–relevant *Atp13a3* variant develop PAH. This suggests that disrupted polyamine homeostasis represents a new genetic mechanism promoting PAH pathobiology.

ATP13A3 most likely functions as a regulator of cellular polyamine content from recycling endosomes.^14,15,24^ This function mirrors the closely related ATP13A2, a late endo/lysosomal polyamine exporter that delivers polyamines into the cytosol, preventing lysosomal accumulation while regulating cellular content.^13^ The expression patterns of *ATP13A2* and *ATP13A3* partly explain their differing disease associations. In mice, *Atp13a2* is widely expressed albeit with higher expression in the brain^25^ and pathogenic *ATP13A2* variants in humans cause an early onset parkinsonism, Kufor–Rakeb syndrome.^26^  *Atp13a3* is also widely expressed in mice, with particularly high expression in liver,^25^ although in humans potentially pathogenic variants may cause PAH.^7^ The widespread expression of *Atp13a2* and *Atp13a3* suggests possible redundancy, but the distinct diseases associated with potentially pathogenic variants in humans suggest some non-redundant roles.

We studied several EC models of *ATP13A3* deficiency, namely transient siRNA, stable miRNA, and patient-derived *ATP13A3*^LK726X^ BOECs in parallel with overexpression experiments. Of note, the mRNA expression of *ATP13A1* and *ATP13A2* was not altered by *ATP13A3* deficiency, suggesting a lack of compensation by these related proteins. We found that *ATP13A3* deficiency in these EC models suppressed the uptake of BDP-labelled polyamines. The reduction in uptake of each polyamine differed between the cell models, possibly reflecting model-specific variations in the receptor-mediated endocytosis pathway proximal to ATP13A3-mediated transport to the cytosol.^17^ We also observed reductions in endogenous polyamine levels in ATP13A3-deficient EC cell models, and an attenuated response to PUT supplementation. Conversely, ATP13A3-WT protein overexpression led to increased uptake of mainly PUT-BDP and SPD-BDP, and a higher intracellular PUT content. The differences observed between labelled polyamine uptake vs. endogenous levels are not unusual. Fluorescently labelled polyamines behave similarly to radiolabelled polyamines, thus directly representing uptake.^24^ In contrast, quantifying endogenous polyamine levels by MS reflects an integration of the polyamine metabolic pathways, so the uptake of one polyamine species may impact all the polyamine species.

In this study, we have also identified that the loss of *ATP13A3* not only reduces intracellular polyamine levels but also leads to a reprogramming of the polyamine metabolism pathway. Despite subtle differences between our EC models, we generally observed increased polyamine biosynthesis (e.g. up-regulated *ODC1/AZIN1* or reduced *OAZ1*) as well as a reduced polyamine catabolism (e.g. lower SAT1 and/or PAO), both of which may compensate for polyamine loss. This probably explains why multiple endogenous polyamine species are simultaneously affected. One limitation of cells isolated from patients with PAH is the potential impact of changes acquired due to the disease state^27^ and the heterozygous expression of the variant compared with the greater reduction achieved with siRNA transfection. These may explain why transcript alterations of polyamine biosynthesis enzymes with short-term *ATP13A3* loss of a large magnitude (siRNA transfection) differed from chronic deficiency (genetic defects). With specific reference to *OAZ1*, a question remains regarding the mechanism by which its expression is reduced in hPAECs and BOECs following *ATP13A3* knockdown. This may be due to a transcriptional response mediated by changes in polyamines either directly, or by activation of other signalling pathways. The regulation of *OAZ1* would be of interest in future studies.

In this context of altered polyamine metabolism, it remains difficult to deduce the precise polyamine transport specificity of ATP13A3 from cellular data. Our observations suggest that ATP13A3 displays a broad polyamine selectivity that exerts the strongest impact on PUT uptake and content. Similar findings were also reported for Atp13a3 in CHO-MG cells, although unlabelled SPD and SPM competed with ATP13A3-dependent PUT-BDP uptake.^14^ However, ATP13A3 loss in pancreatic cancer cells prevented the uptake of radiolabelled SPD and SPM, with little effect on PUT uptake.^15^ Whether the polyamine specificity of ATP13A3 differs in a cell-dependent manner or if the methods of ATP13A3 manipulation exert different effects have yet to be established.

The dual effect of *ATP13A3* deficiency on polyamine uptake and polyamine homeostasis in ECs may explain the strong impact of *ATP13A3* reduction on endothelial health and functionality. Polyamines are essential for cell growth,^16,17^ with polyamine depletion leading to cell cycle arrest.^17^  *ATP13A3* deficiency suppressed serum-dependent proliferation and *cyclin A*, *E*, and *B* mRNA expression in BOECs implying repression of G1-S transition and DNA synthesis in the cell cycle. Polyamine depletion may promote cell apoptosis in response to pathogenic insults.^28,29^ We show that *ATP13A3* deficiency increases apoptosis in hPAECs, BOECs, and *ATP13A3*^LK726X^ BOECs when they are stressed by serum starvation, an effect that is rescued with ATP13A3-WT overexpression. Moreover, polyamines are essential for epithelial cell–cell junctions,^30^ and we observed that *ATP13A3* deficiency exacerbated thrombin-dependent hPAEC monolayer permeability, indicating an important role of *ATP13A3* in maintaining endothelial integrity.

Using our EC models, we examined the functional impact of PAH-associated missense variants using lentiviral overexpression. A homozygous V855M variant was identified in a child with early onset PAH leading to early death.^31^ The other PAH missense variants (L675V, M850I, R858H, and L956P) were heterozygous and found in older patients with PAH.^7^ Collectively, our data confirm pathogenicity due to a loss of function, although possibly by differing impacts on ATP13A3 protein function, negatively impacting on (i) protein expression, (ii) transport activity, (iii) polyamine homeostasis, and/or (iv) substrate specificity. These differences may relate to cell type (e.g. for M850I), transient vs. the lentiviral stable variant expression (the latter being more potent potentially increasing the window), and/or the possible stabilizing effect of the GFP tag (for V855M and R858H). The latter may explain the low protein stability of untagged V855M and R858H variants compared with GFP fusion products. Furthermore, the variable impacts of these ATP13A3 variants may suggest other modifiers are required to promote disease progression. Interestingly, interferon-β (IFN-β) therapy induced PAH in a multiple sclerosis patient with a nonsense *ATP13A3* variant (Glu514*) and IFN-β withdrawal improved their PAH symptoms.^32^

Combined, our results convincingly show that the disease-associated *ATP13A3* variants present a loss of function, which based on our knockdown studies, would have a major impact on polyamine uptake and homeostasis in ECs. Although many genes disrupted in PAH tend to be enriched in the endothelium, *ATP13A3* mRNA is expressed at similar levels in hPAECs, BOECs, and human pulmonary artery smooth muscle cells.^7^ Given that pulmonary artery smooth muscle cell proliferation is a hallmark of medial thickening in PAH, future investigations into the impact of *ATP13A3* disruption on smooth muscle cell function would be of interest.

Importantly, mice harbouring a heterozygous *Atp13a3*^P452Lfs^ variant associated with human PAH developed increased RVSP and RV hypertrophy without altered systemic blood pressure. Intriguingly, this was only observed in male mice aged to 6 months, while female mice were unaffected. This does not reflect the female predominance reported previously in patients with PAH,^7^ though it is not known if human males with pathogenic *ATP13A3* variants die at a young age. Alternatively, there may be sex differences in the susceptibility of humans and mice to pathogenic *ATP13A3* variants. In addition to elevated RVSP in 6-month-old male mice, analysis of the lung vasculature revealed a significantly higher percentage of fully muscularized small pulmonary arteries, suggesting that genetic deficiency of *Atp13a3* leads to the development of PAH by affecting small pulmonary vessels. Although the increase in pressure in heterozygous *Atp13a3*^P452Lfs^ variant mice was modest compared with the large pressure increases seen in hypoxia- or Sugen/hypoxia-induced mouse PAH models, the PAH phenotype in *Atp13a3*^P452Lfs^ variant mice arose spontaneously and reproducibly without requiring a stimulus. This observation is not akin to the reduced penetrance observed in genetic mouse models of *Bmpr2* deficiency, which represents the major cause of PAH in humans.^33–35^ In future, it would be interesting to explore the impact of disease-promoting stimuli on PAH and the polyamine pathway in *Atp13a3*^P452Lfs^ variant mice.

In a broader disease context, polyamine dysregulation has been implicated in non-genetic rodent models of PH and human PAH. Excessive lung polyamine accumulation was reported in rats with PH induced by either chronic hypoxia^36,37^ or monocrotaline (MCT).^38–40^ However, the different rodent models appear to be associated with different mechanisms of polyamine accumulation. In MCT rats, increased activity of the synthetic enzymes, ODC and AMD, suggests higher polyamine biosynthesis rates.^38,39^ Administration of the irreversible ODC inhibitor, DFMO, attenuated the increase in mean pulmonary arterial pressure (mPAP).^40^ Conversely, in hypoxic PH, ^14^C-SPD accumulation was elevated in lung tissues,^41^ suggesting that the rates of uptake were increased. Altered polyamine metabolism has also been documented in patients with PAH. Metabolomic analysis has reported increased ornithine and PUT in lung tissues from patients with PAH^42^ and elevated plasma 4-acetamidobutanoate and *N*-acetyl-PUT have been reported in patients with idiopathic/heritable PAH.^43^ Recently, ODC mRNA expression was shown to negatively correlate with mPAP in patients with PAH.^44^ Although abnormal polyamine levels are observed in PAH, it is unclear whether this represents changes in the intracellular or extracellular environment and whether various cell types may exhibit different responses. At this juncture, the mechanisms linking polyamine dysregulation and the pathobiology of PAH have not been clarified.

In conclusion, we have demonstrated that ATP13A3 functions as a polyamine transporter and has a functional role in endothelial homeostasis. PAH-associated variants exhibited impaired ATP13A3-mediated polyamine transport, contributing to disease-associated cellular phenotypes. These findings shed light on the pathogenic mechanism of *ATP13A3* genetic defects leading to a loss of function in PAH and provide new insight into a potential role for polyamine dysregulation in the pathobiology of PAH.

**Translational perspective**

Rare missense *ATP13A3* disease–associated variants have been identified in patients with pulmonary arterial hypertension (PAH), although their pathogenicity has not been confirmed. In this study, we show that disease-associated variants hamper the ATP13A3 transport function. ATP13A3 deficiency impairs polyamine homeostasis and uptakes and drives endothelial dysfunction. Conversely, overexpression increases polyamine uptake and rescues the proapoptotic phenotype of cells harbouring a disease-associated variant. Mice heterozygous for a disease-associated *Atp13a3* variant spontaneously develop PAH. These findings support the rationale for exploring dysregulated polyamine homeostasis in PAH and suggest a potential for therapeutic targeting of polyamine pathways in PAH.

## Supplementary Material

cvae068_Supplementary_Data

## Data Availability

The data are available from the corresponding author by request.
