# Supplementary material for: ATP13A3 variants promote pulmonary arterial hypertension by disrupting polyamine transport
Source: Cardiovasc Res. 2024 Apr 16;120(7):756–68. doi: 10.1093/cvr/cvae068 (PMC11135649; doi:10.1093/cvr/cvae068)

**­­ONLINE SUPPLEMENT**

***ATP13A3* Variants Promote Pulmonary Arterial Hypertension by Disrupting Polyamine Transport**

Bin Liu^1*^, Mujahid Azfar^2*^, Ekaterina Legchenko^1*^, James A. West^3, 4, 5^, Shaun Martin^2^, Chris Van den Haute^6, 7^, Veerle Baekelandt^6^, John Wharton^8^, Luke Howard^8^, Martin R. Wilkins^8^, Peter Vangheluwe^5†^, Nicholas W. Morrell^1†^ and Paul D. Upton^1†#^

^*^ joint first author, ^†^ joint senior authors

1 Section of Cardio and Respiratory Medicine, Department of Medicine, Cambridge, UK, 2 Department of Cellular and Molecular Medicine, KU Leuven, Leuven, Belgium, 3 Cambridge Institute of Therapeutic Immunology and Infectious Disease, Jeffrey Cheah Biomedical Centre, Cambridge, UK 4 Division of Gastroenterology and Hepatology, Department of Medicine, Cambridge, UK, 5 Department of Biochemistry and Cambridge Systems Biology Centre, University of Cambridge, Cambridge, UK 6 Laboratory for Neurobiology and Gene Therapy, Department of Neurosciences, KU Leuven, Leuven, Belgium 7 Leuven Viral Vector Core, KU Leuven, Leuven, Belgium 8 Faculty of Medicine, National Heart & Lung Institute, Imperial College, London, UK.

**^#^ Corresponding author:** Dr Paul D Upton PhD, Department of Medicine, Heart and Lung Research Institute, Papworth Road Cambridge CB2 0BB, UK, pdu21@medschl cam.ac.uk

**Online Supplementary Methods**

**Analysis of the pulmonary vasculature.** Pulmonary arteriolar muscularization was assessed on sections of fixed mouse lung tissue (3.5 μm thick) labelled with monoclonal mouse anti–smooth muscle α-actin (αSMA) (clone 1A4; Dako) antibody, followed by polyclonal goat anti-mouse horseradish peroxidase. To detect staining, the ARK kit (Dako) was used in accordance with the manufacturer’s instructions. Antibody staining was visualized using 3-3 diaminobenzidine hydrochloride as substrate-chromogen and counterstained with Carrazzi’s hematoxylin. Pulmonary arteriolar muscularization was assessed by identifying alveolar ducts and categorizing the accompanying intra-acinar artery as non-muscularized, partially muscularized, or fully muscularized by the degree of SMA immunostaining. A minimum of 20 vessels with diameters ranging from 25 to 75 μm were categorized per animal.

Invasive hemodynamic measurements as well as morphometric analyses were performed on randomly picked mice and the experimenter was blinded regarding the genotype.

**Wheat germ agglutinin (WGA) staining of heart tissues.** Formalin-fixed paraffin-embedded tissue sections **(**3µm thick) were deparaffinised and rehydrated. The sections were permeabilised with 0.3% Triton in PBS for 20 minutes at RT, blocked with 3% BSA in PBS for another 20 minutes. The tissue sections were then incubated with WGA (1:50, W21404, Invitrogen) in 3%BSA in PBS for 1 hour at RT in the dark. The slides were then mounted with Vectashield HardSetTM Antifade Mounting Medium with DAPI (Vector Laboratories, USA) and visualised using a DMi8 fluorescence microscope (Leica, Germany).

**Picrosirius red staining for collagen deposition.**Formalin-fixed paraffin-embedded heart sections were deparaffinised, rehydrated and stained according to standard protocols. Complete section slides were photographed at 200X magnification (Zeiss AxioScan, Germany). Images of the zoomed in RV (11-20 images per animal) were taken for each heart. Fibrosis was determined as the amount of red staining in each RV image and expressed as a percentage of the total area of the image. The mean percentage of red-positive area was reported for each RV.

**Transient transfection with siRNA.** Cells were maintained in Opti-MEM-I reduced serum media (Invitrogen) for 2 h prior to the addition of DharmaFect1™ (Dharmacon, GE) transfection reagent (4 µl/well in a 6-well plate) with or without siRNA for *ATP13A3* (SASI_Hs02_00356805) or ON-TARGETplus non-targeting Control Pool (siCP) (GE Dharmacon) at a final concentration of 10 nM. The cells were incubated with the siRNA/DharmaFECT1™ for 4 h at 37℃ before the transfection media were replaced with full growth media. Cells were kept in growth media for 24 h before further treatment. Knockdown efficiency was confirmed by assessing mRNA expression RT-qPCR or immunoblotting.

**Transient plasmid DNA transfection**. Prior to transfection, HMEC-1 cells were seeded at a density of 250,000 cells/well into a 6-well plate and allowed to adhere overnight. Cells were then incubated in Opti-MEM-I reduced serum media (Invitrogen) for 3 h prior to transfection with 1 µg of pcDNA6.2 expression plasmids encoding either wild type or PAH-associated variant (L675V, M850I, V855M, R858H, L956P) *ATP13A3* with an N-terminal GFP tag. Plasmids were transfected using 9 μl /reaction Lipofectamine LTX with 2.5 μl /reaction Plus reagent (Thermo Fisher Scientific). Cells were incubated with lipoplexes at 37°C for 4 h before being returned to HMEC-1 full growth media. Twenty-four hours post-transfection, cells were trypsinised and reseeded into collagen-coated 4-chambered Nunc™ Lab-Tek™ II Chamber Slides™ (Thermo Fisher Scientific) for immunostaining or other experimental purposes.

**Lentiviral transduction.** Lentiviral vectors encoding wild type human *ATP13A3*, the artificial transport dead D498N mutant (D498N) or PAH-associated variants (L675V, M850I, V855M, R858H, L956P) were generated by triple transduction of a transfer plasmid (pCHMWS-ires-puro), an 8.91 packaging plasmid, and a VSV-G envelope plasmid as previously described. ^1^ Prior to transduction, hPAECs were seeded into 6-well plates at a density of 200,000 cells/well and allowed for attaching overnight. The following day, cells were transduced with the lentiviral vectors diluted in EGM-2 supplemented with 2% FBS. hPAECs were transduced with lentiviral particles for 72 h for the optimal transduction. Cells were then used for the following functional assays or lysed directly for extracting protein or RNA.

For stable over-expression of the un-tagged PAH-associated variants, wild type human ATP13A3 or the artificial D498N mutant via lentiviral transduction in HMEC-1 cells, different vector titres were used. Expression was confirmed using immunoblotting and only cells with comparable expression were used. Transduced cells were kept under puromycin (Sigma) selection at the final concentration of 2 µg/mL.

**Stable knockdown of *ATP13A3.*** Stable *ATP13A3* knockdown HMEC-1 cell lines were generated using microRNA (miR) based short-hairpin lentiviral vector transduction. Lentiviral particles were produced as described previously ^1^. For the viral transduction, HMEC-1 cells were seeded in a 24-well plate at a density of 100,000 cells/well after which they were incubated with the viral vectors for up to 72 h. Knock-down viral vectors directed at four different target sequences were validated after which the three most potent sequences were selected for further experiments: miR2: AATCACAACAGATTCGTTATTT; miR3: TCAATCGTAAGCTCACTATATT; miR4: AGACCACCTTCGGGTCTTATAT with miR-Fluc: ACGCTGAGTACTTCGAAATGTC used as a negative control. Following transduction, the cells were subjected to selection using Blasticidin (InvivoGen, ant-bl-1) at a concentration of 5 µg/mL.

**Genotyping of the *ATP13A3*^LK726X^ blood outgrowth endothelial cells.** *ATP13A3*^LK726X^ BOECs and control BOECs (C4, C7, C35) were grown in 6-well plates at a density of 200,000 cells per well overnight. Genomic DNA (gDNA) of the cells was extracted using the DNeasy Blood & Tissue Kit (Qiagen, West Sussex, UK) in accordance with the manufacturer’s instructions. The resultant gDNA was further PCR amplified with primers (FORWARD: 5’-TGGTTCTTGTGTCACATTTTCAGG-3’; REVERSE: 5’-ACACTCCATTTGCTTCTGTGT-3’) using the AccuPrimeTM Pfx DNA Polymerase kit. PCR products were purified with the Invisorb® Fragment CleanUp kit (Stratec Molecular, Germany) before sanger sequenced (GENEWIZ, UK).

**Proliferation Assay.** Cells were seeded in 24-well plates at a density of 30,000/well and left to adhere overnight. Transfection of si*ATP13A3,* siCP or DharmaFECT1™ reagent alone was then performed and cells returned to full growth media afterwards. For assessment of hPAEC proliferation, cells were quiesced in EBM-2/0.1% FBS for 8 h before culturing in EBM-2 media containing 2% FBS (v/v) for six days. Treatments were replenished every 48 h. On day 6, cells were trypsinised with 150 μl/well 0.5% trypsin (Sigma-Aldrich) and quenched with 60 μl/well of the relevant growth media, followed by 90 μl/well of trypan blue (0.4%, Sigma-Aldrich). All 300 μl of cell suspension was transferred into a newly labelled Eppendorf tube, and cells were counted using a haemocytometer. To establish if the level of knockdown was retained, RNA was collected from hPAECs on day 0 (48 h post-transfection) and day 6 followed by assessing *ATP13A3* mRNA expression by qPCR.

**Apoptosis assay.** To assess the caspase-3.7 activities, cells were seeded at a density of 150,000/well into 6-well plates and transfected with si*ATP13A3* (Sigma-Aldrich)*,* siCP (GE Dharmacon) or DharmaFECT1™ (GE Dharmacon) alone. For each condition, cells were trypsinised from 6-well plates, reseeded in triplicates into a 96-well plate at a density of 15,000-20,000/well and left to adhere overnight. Cells were quiesced in EBM-2/0.1% FBS for 24 h before culturing in EBM-2/0.1%FBS for 16 h. For measuring caspase-3/7 activities, 100 μl Caspase-Glo® 3/7 Reagent (G8091 Promega) was added into each well, then incubated and mixed on a plate shaker in the dark for 15 min at room temperature. The entire 200 μl from each well was transferred into the corresponding well of a white-walled 96-well plate and luminescence was read in a GloMax® luminometer (Promega).

**Endothelial permeability assay.** This assay measures the transit of horseradish peroxidase (HRP) across endothelial monolayers seeded in transwell inserts. hPAECs subjected to siRNA transfection were trypsinised and reseeded at a density of 50,000 cells/insert into Corning® Transwell® chambers with polyester membrane cell culture inserts (Corning) and allowed to attach for 30 min before adding 1ml of EGM2 supplemented with 2% FBS (Promocell) to the lower 24-well plate chamber. hPAECs were incubated overnight and then serum-starved for 6 h by replacing the media in the inserts with 200 μl, and the bottom chambers with 1ml of EBM2/0.1% FBS. Following serum-starvation, media were then replaced with 2% FBS supplemented EBM2 with or without the addition of 1 U/ml Thrombin (Sigma) and incubated for 1 h at 37°C. In the meantime, 0.05 M Phosphate Citrate Buffer was prepared by dissolving one capsule of Phosphate-Citrate buffer with Sodium Perborate (Sigma) into 100 ml of PBS. The o-Phenylenediamine dihydrochloride (OPD) developing solution was prepared by adding one OPD (Sigma) tablet into 50 ml Phosphate-Citrate buffer. Prior to the assessment of permeability, media in the inserts was replaced with 100 μl of EBM2 supplemented with 2% FBS containing 25 nM HRP(Sigma) with or without the addition of 1 U/ml Thrombin. 3 x 15 μl medium from the lower chambers were collected into a new 96-well plate at the following time points: 0min, 15min, 30min, 1 h, 1.5 h and 2 h. 150 μl/well of OPD buffer was then added into the 96-well plate and the absorbance measured at 490 nm over 10 to 20 min.

**Cytotoxicity Assay**:

Cells were seeded into 96 clear-well F-bottom plates at a cell density of 2.5 X 10^4^ cells per well and incubated overnight (5% CO2 and at 37°C) to attach to the bottom of the wells. Doses of the screened compounds were made in DMEM cell culture medium and 100 μL of the dilutions were added to the corresponding wells. After an overnight incubation, the cells were washed twice with PBS. Finally, 50 μL of 4-Methylumbelliferyl heptanoate (MUH reagent) (Sigma), dissolved in PBS to a final concentration of 100 μg/mL, was added per well. The cells were then incubated at 37°C in the dark for 45 min. The fluorescence was measured using a multi-mode plate reader (Flexstation3, Molecular Devices) with excitation at 355 nm, emission at 460 nm and cut-off at 455 nm. Results were normalised to the untreated control.

**RNA extraction and quantitative reverse transcription-PCR.** Total RNA was extracted using RNeasy Mini Kit buffers (Qiagen, West Sussex, UK) and Silica Membrane Mini Spin Columns (EconoSpin) following the manufacturer’s instructions. Equal amounts of RNA (~1 μg) were then reverse transcribed into cDNA using a High Capacity Reverse Transcriptase kit (Applied Biosystems). 2 μl cDNA, 1.8 μl associated premixed primer sets (final concentration in mix = 200nM), 5 μl 2X SYBR Green JumpStart Taq ReadyMix (Sigma-Aldrich), 0.2 μl ROX reference dye (Invitrogen) and 1 μl DEPC-treated water were prepared into one well of a MicroAmp® Optical 384-Well Reaction Plate (Applied Biosystems) before loading on a QuantStudio 6 Flex Real-Time PCR System (Applied Biosystems). Amplification reactions were initiated with a 2-min pre-incubation at 95°C, followed by 50 amplification cycles of 30-second denaturation at 95°C, 30 seconds annealing at 55°C and 30-seconds extension at 72°C. Melt curve analysis was performed to rule out nonspecific amplification, and no-template controls were included. Primers for human genes (supplement table 3) were designed using Primer-BLAST (http://www.ncbi.nlm.nih.gov/tools/primer-blast/), and primer efficiency confirmed before use. The relative expression levels of target genes were calculated using the 2^^-(△△Ct)^ method by normalizing to the stably expressed housekeeping genes, Beta-2-Microglobulin (*B2M*) or β-actin (*ACTB*). Differences in gene expression are presented as the fold change relative to control. Relative expression of target genes in different cell lines or mouse tissues was determined as 2^^-(CTtarget -CThousekeeping)^.

**Cellular polyamine measurement**

**Extraction of aqueous metabolites.** Following siRNA transfection or lentiviral transduction, cells were washed twice with phosphate-free buffer (162 mM ammonium acetate 7.4) before being lysed with 4:1 methanol: water lysis buffer. The resulting cell lysate was sonicated for 5 min in a water bath sonicator followed by an additional 5-min sonication if obvious cell debris was still present. The residual debris was then removed by pelleting the cell homogenate at 21000 x g for 10 min at RT, and the supernatant was carefully transferred into new tubes.

**LC-MS sample preparation for analysis of polyamines.** Aqueous extracts of cells were dried using a centrifugal evaporator (Savant, ThermoFisher) and reconstituted in 50 µl of 10 mM ammonium acetate containing 2 µM [^13^C_10_, ^15^N_5_] adenosine monophosphate, 10 µM succinic acid [^13^C10], 10 µM d8 putrescine and a 1 in 5000 diluted [U^13^C_10_, U^15^N_5_] mixture of amino acids (all purchased from Sigma Aldrich with the exception of the d8 putrescine which was obtained from CDN isotopes) as internal standards. The resulting solution was vortexed then sonicated for 5 min. followed by brief pulsed centrifugation to recover the maximum volume. After centrifugation the supernatant was transferred with an automatic pipette into a 300 µl glass vial (Chromacol) and capped (Agilent) ready for analysis.

**LC-MS analysis of polyamines.** For all analysis a Q Exactive Plus orbitrap coupled to a Vanquish Horizon ultra-high performance liquid chromatography system was used. LC analysis was carried out using an ACE Excel C18-PFP column (150 × 2.1 mm, 2.0 µm, Hichrom). Mobile phase A consisted of water with 0.1% formic acid with 10 mM ammonium formate and mobile phase B was acetonitrile with 0.1% formic acid. For gradient elution mobile phase B was held at 0% for 1.6 min followed by a linear gradient to 30% B over 4.0 min, a further increase to 90% over 1 min and a hold at 90% B for 1 min with re-equilibration for 1.5 min giving a total run time of 6.5 min. The flow rate was 0.5 mL/min and the injection volume was 2 µL. The needle wash used was 1:1 water:acetonitrile.

Samples were run using electrospray ionisation in positive ion mode only. Source parameters used for the orbitrap were a vaporizer temperature of 450°C and ion transfer tube temperature of 320°C, an ion spray voltage of 3.5 kV and a sheath gas, auxiliary gas and sweep gas of 55, 15 and 3 arbitrary units respectively with an S-lens RF (radio frequency) of 50%. A full scan of 60-900 m/z was used in positive ion mode at a resolution of 70,000 ppm.

**LC-MS data processing.** Data were acquired, processed and integrated using Xcalibur (Version 4.1, Thermo Fisher Scientific). Retention times and accurate masses for putrescine, acetyl putrescine, spermine and spermidine were validated against injections of known external standards (obtained from Sigma Aldrich). Peak areas corresponding to metabolite levels were manually quantified and normalised to the appropriate internal standard and presented as relative areas.

**Immunoblotting:** Cells were snap-frozen and lysed in SDS-lysis buffer (125mM Tris (pH 7.4), 2% SDS, 10% glycerol) containing an EDTA-free protease inhibitor cocktail (Roche) or by using RIPA buffer (Thermo Fisher) containing protease inhibitors (Sigma). Protein concentrations were assessed using a modified Lowry assay (Bio-Rad DC Assay, Bio-Rad) or Pierce BCA Protein Assay Kit (Thermo Fisher). Primary antibodies for ATP13A3 (amino acids 488-631, Sigma, HPA029471). and GAPDH (Sigma, G8795) were dissolved at a dilution of 1:1000 and 1:5000 respectively in 1% BSA/TBS-tween and incubated overnight at 4°C. The following secondary antibodies, IgG HRP-linked Anti-rabbit (Cell Signalling, 7074S) and IgG HRP-linked Anti-mouse (Cell Signalling, 7076S), were dissolved in 5% milk/TBS-tween solution at a dilution of 1:5000 for 1 h, Bands were detected using SuperSignal™West Pico PLUS chemiluminescent Substrate (Thermo Fisher) or incubated with enhanced chemi-luminescence reagent (GE Bioscience) and visualised by on the Bio-Rad Chemidoc ™ MP imaging system or using X-ray film (GE healthcare)..

**Immunofluorescence staining:** Prior to cell seeding, 4-chambered Nunc™ Lab-Tek™ II Chamber Slides™ (Thermo Fisher Scientific) were pre-coated with 500μl/chamber Type I Rat Tail Collagen (BD Biosciences) for 90 min before washing three times with PBS. Following siRNA or plasmid DNA transfection, cells were trypsinised and reseeded into collagen-coated 4-chambered slides and left to adhere overnight. Cells were washed with PBS and fixed in 4% (v/v) Paraformaldehyde (Sigma) at RT for 10 min, then permeabilised with 0.05%(v/v) Saponin in 0.5% BSA/PBS for 20 min and blocked in 0.5% BSA/PBS for 1 h. The chambers were removed and the slides incubated overnight with the primary antibodies listed below (supplement table 4). Cells were then washed three times with PBS and incubated with the corresponding fluorescent antibodies (supplement table 4) at RT for 1 h. Cells were then washed twice with PBS before mounting with Vectamount™ medium containing DAPI (Vector Laboratories) and imaging on a Leica Sp5 confocal microscope platform (Leica microsystem).

**Confocal Microscopy of Cellular BODIPY-PUT Uptake:**

Cells were seeded in a 12-well plate at a density of 10,000 cells/well on coverslips. The following day, they were then then treated with the polyamine-BODIPY probes as described earlier. The medium was then aspirated, the cells washed with PBS and fixed with 4% paraformaldehyde for 30 min at 37°C after which the cells were once again washed with PBS. Next the cells were permeabilized with 0.1% Triton-X and blocked for 1 h with 0.1 M glycine and then for 1 h in PBS containing 0.1% Tween (PBS-T), 10% FBS and 1% BSA. Next the cells were stained with DAPI for 15 min, the slides were washed with PBS-T and glued onto slides using the Alexa FluorSave reagent and left to dry. Slides were imaged using the LSM780 confocal microscope.

**REFERENCES**

1. Osório L, Gijsbers R, Oliveras-Salvá M, Michiels A, Debyser Z, Van den Haute C, Baekelandt V. Viral vectors expressing a single microRNA-based short-hairpin RNA result in potent gene silencing in vitro and in vivo. *J Biotechnol* 2014;**169**:71-81.

**Online supplementary figures and tables**

**
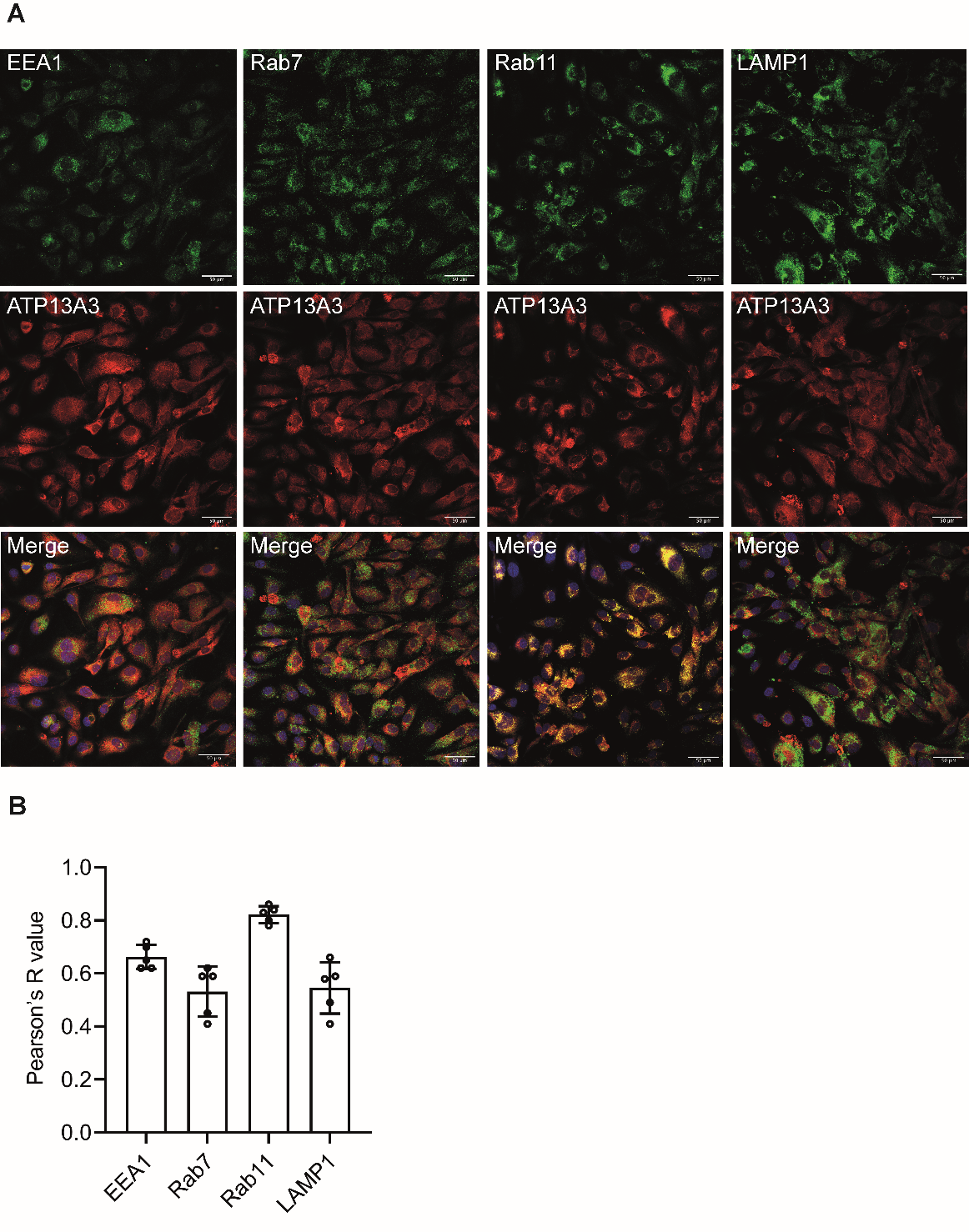
**

**Supplement Figure 1. Analysis of endogenous ATP13A3 cellular compartmentalisation in HMEC-1 cells. (**A) Confocal images (40X, scale bar = 10 µm) of HMEC-1 co-stained with anti-ATP13A3 and antibodies against either EEA1, Rab7, Rab11 or LAMP1. (B) Pearson’s coefficients of ATP13A3 to different endosomal markers in HMEC-1.

**
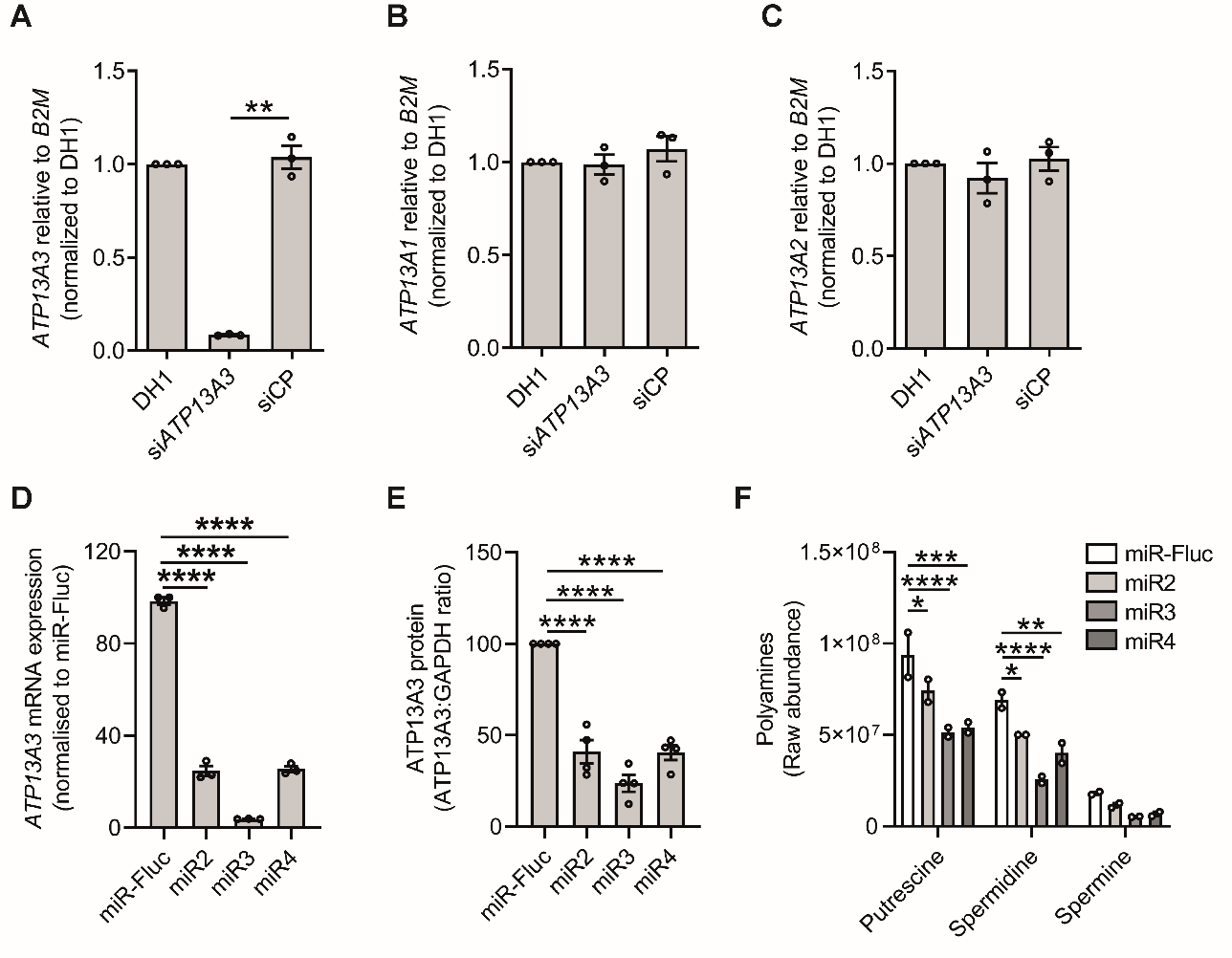
 Supplement Figure 2. mRNA expression of P5-type ATPases in *ATP13A3*** **silenced hPAECs and HMEC-1 cells** Expression of (A) *ATP13A3* (B) *ATP13A1* (C) *ATP13A2* mRNAs in hPAECs transfected with DharmaFECT1™ (DH1) alone, si*ATP13A3* or non-targeting siRNA control (siCP) (n=3 experiments). (D,E) *ATP13A3* (D) mRNA and (E) protein expression in HMEC-1 cells stably expressing miR2-4 and miR-Fluc. Data (n=3 experiments) are mean ± SEM expressed relative to (A-C) DH1 or (D,E) miR-Fluc. (F) Metabolomic analysis of the raw abundance of basal intracellular putrescine, spermidine and spermine levels in HMEC-1 cells stably expressing miR2-4 and miR-Fluc (n=2 experiments). Data were analysed using a One-way ANOVA with Tukey’s *post hoc* test for multiple comparisons. **P<0.05, ***P<0.01, ****P<0.0001.





**Supplement Figure 3. Schematic of cellular polyamine metabolism pathways.** Arginase 1 (ARG1) mediates the conversion of arginine to ornithine, which is then converted to putrescine by ornithine decarboxylase (ODC). Putrescine is then converted to spermidine by spermidine synthase (SRM), which can then be converted to spermine by spermidine synthase (SMS). Conversely, spermine is converted to spermidine by spermine oxidase (SMO/SMOX), spermine/spermidine acetyltransferase (SAT1) and polyamine oxidase (PAO), the latter two also mediating conversion of spermidine to putrescine. In addition, S-adenosylmethionine (AMD1) activity can generate spermidine or spermidine via decarboxylaton of S-adenosyl-methionine to the intermediate, methyl-S-adenosylthiopropylamine (DC-SAM), which can be converted to spermidine by SRM.


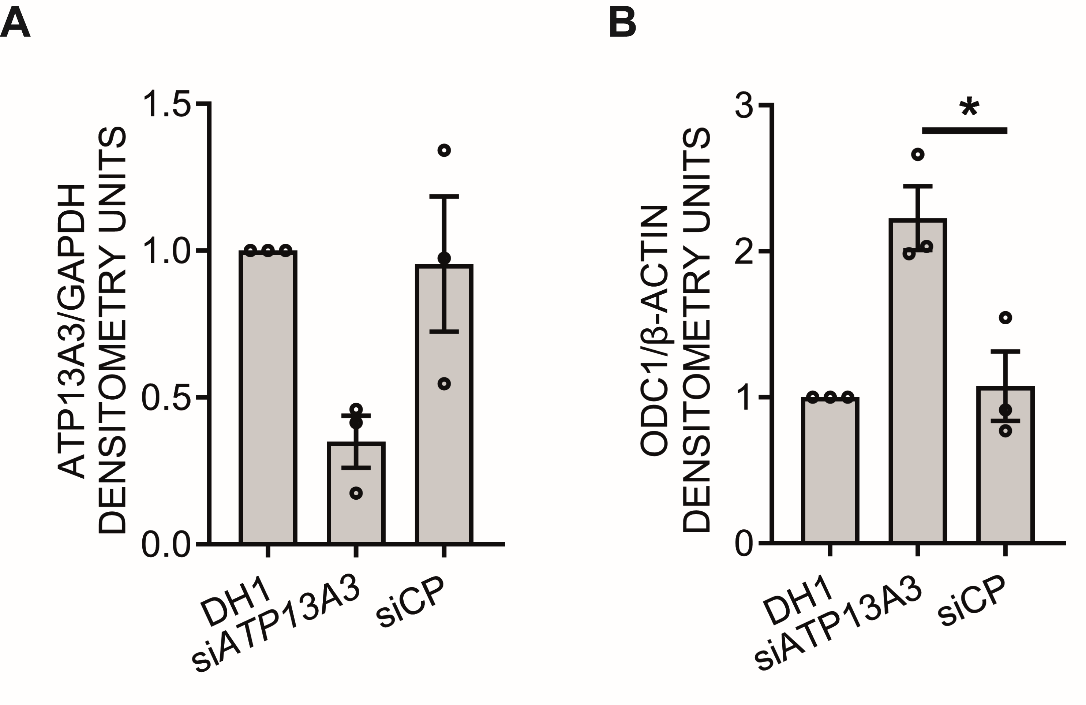


**Supplement Figure 4.** Densitometric analysis of (A) ATP13A3 and (B) ODC normalised relative to α-tubulin and β-actin respectively and as fold-change relative to DH1. (Blots are shown in Figure 3A). Data analysed with an unpaired t-test, *P<0.05


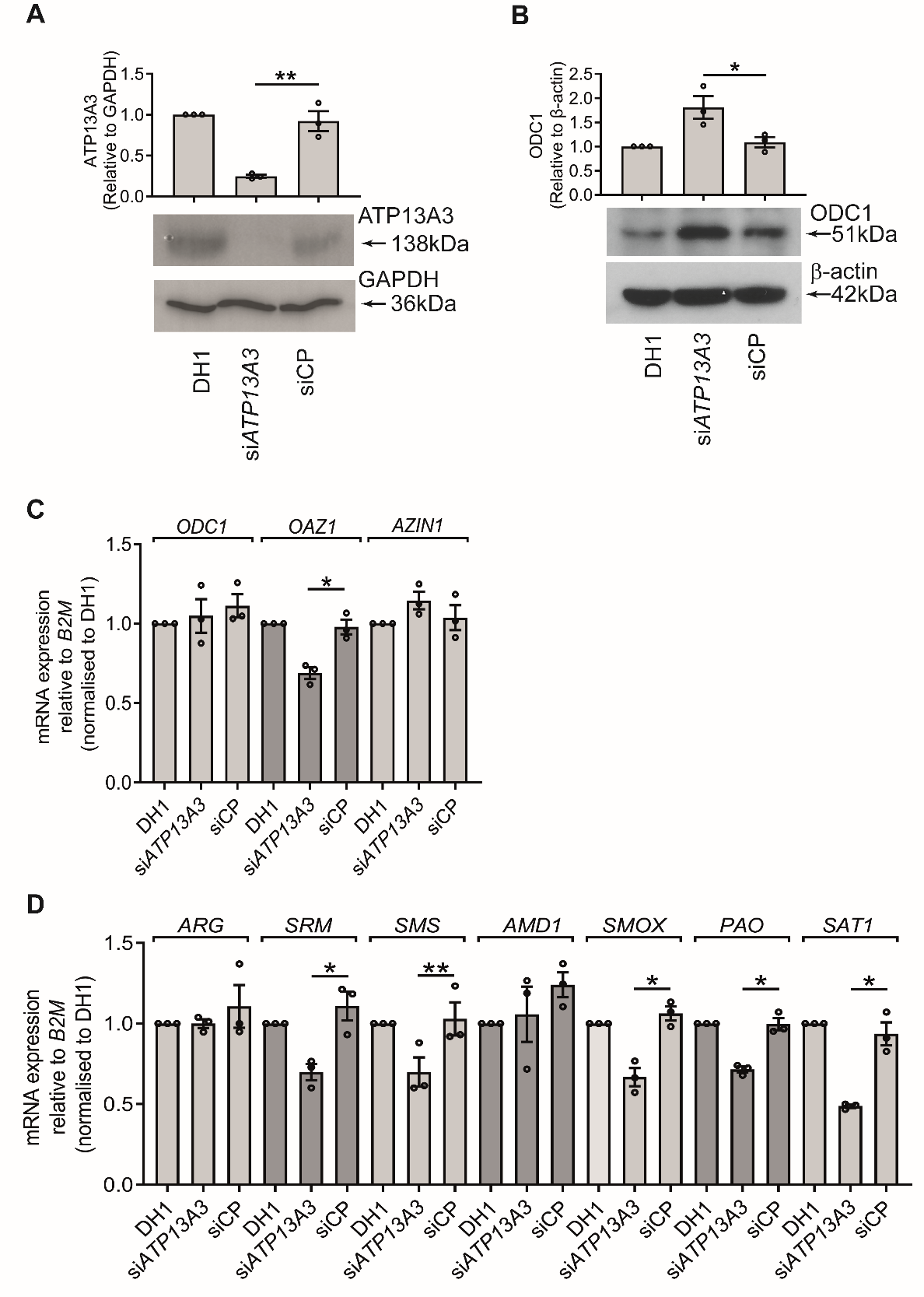


**Supplement Figure 5. *ATP13A3* silencing disrupted polyamine metabolism in BOECs** Immunoblotting of (A) ATP13A3 and (B) ODC1 in control BOECs transfected with DharmaFECT1™ (DH1) alone, si*ATP13A3* or non-targeting siRNA control (siCP). Densitometric analysis of ATP13A3 and ODC relative to α-tubulin and β-actin respectively are shown above the blots. (C,D) Transcriptional alteration of (C) *ODC1, OAZ1* and *AZIN1,* (D) catabolic enzymes (*SMOX*, *PAO*, *SAT1*) mRNA and (E) polyamine biosynthesis enzymes (*ARG1*, *SRM*, *SMS*, *AMD1*) in BOECs transfected with DH1, si*ATP13A3* or siCP. Data (n=3 experiments) are mean ± SEM and are fold-change relative to DH1. Data were analysed using a One-way ANOVA with Tukey’s post hoc test for multiple comparisons. *P<0.05, **P<0.01 and ***P<0.001

**

**

**Supplement Figure 6. Effects of supplementation with polyamines on hPAEC proliferation.** Proliferation of hPAECs following 6-day incubation in EBM2 with 2% alone, or supplemented with (A) putrescine, (B) spermidine or (C) spermine, with or without DFMO at the indicated concentration. Media were replenished every other day. (D) hPAECs transfected with DharmaFECT1™ (DH1), si*ATP13A3* or siCP were treated with 10µM of either putrescine (PUT), spermidine (SPD) or spermine (SPM) and assessed for proliferation at day 6. Data for (A-C) are presented as fold change relative to untreated control and (D) normalised to untreated DH1. Mean ± SEM are shown and data were analysed by one-way ANOVA with Tukey’s post hoc test for multiple comparisons.

**
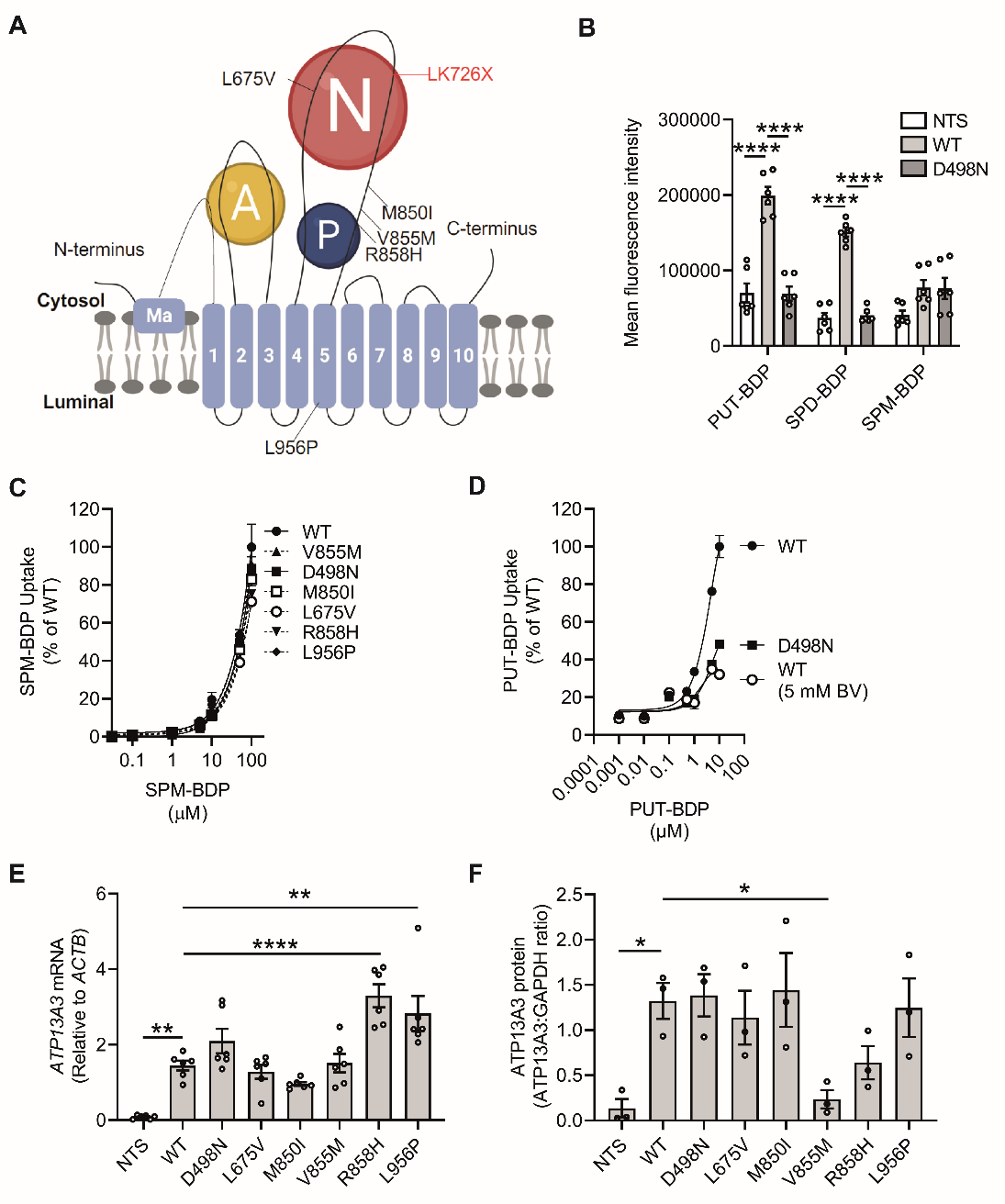
**

**Supplement Figure 7. ATP13A3 expression and validation of WT-ATP13A3 as a polyamine transporter. (**A) Predicted ATP13A3 topology showing the three cytosolic domains, the Actuator domain (A, yellow), nucleotide domain (N, red) and the phosphorylation domain (P, blue). The protein contains 10 membrane-spanning segments, responsible for substrate binding and provision of structural support. An additional N-terminal segment (Ma) is embedded in the membrane. The location of disease related missense (black) and frameshift (red) variants are indicated on this structure. (B) Flow-cytometry analysis (n=3 experiments, two technical replicates per experiment) of PUT-BDP, SPD-BDP and SPM-BDP uptake after 30 minutes exposure in HMEC-1 cells stably expressing ATP13A3 wild-type (WT) or the artificial D498N mutant compared to non-transduced (NTS) cells. (C) Flow-cytometry analysis for assessment of cellular uptake increasing concentrations of SPM-BDP (n=2 experiments) after 30 minutes exposure. Data are normalised to WT. (D) PUT-BDP uptake by HMEC-1 stably overexpressing ATP13A3 WT and D498N protein and in the WT cells treated with 5 mM Benzyl Viologen, a polyamine transport inhibitor. (E,F) The expression of *ATP13A3* (E) mRNA (n=3 experiments) and (F) protein (n=3 experiments) in non-transduced (NTS) HMEC-1 cells compared to those stably expressing untagged ATP13A3 WT (WT), an artificial transport dead mutant (D498N) or five PAH-associated variants (L675V, M850I, V855M, R858H and L956P). (F) mRNA levels are expressed relative to *ACTB* and (G) protein levels were normalised to GAPDH (see Figure 5A). Mean ± SEM are shown and data were analysed by one-way ANOVA with Tukey’s post hoc test for multiple comparisons. *P<0.05, **P<0.005 and ****P<0.0001

**
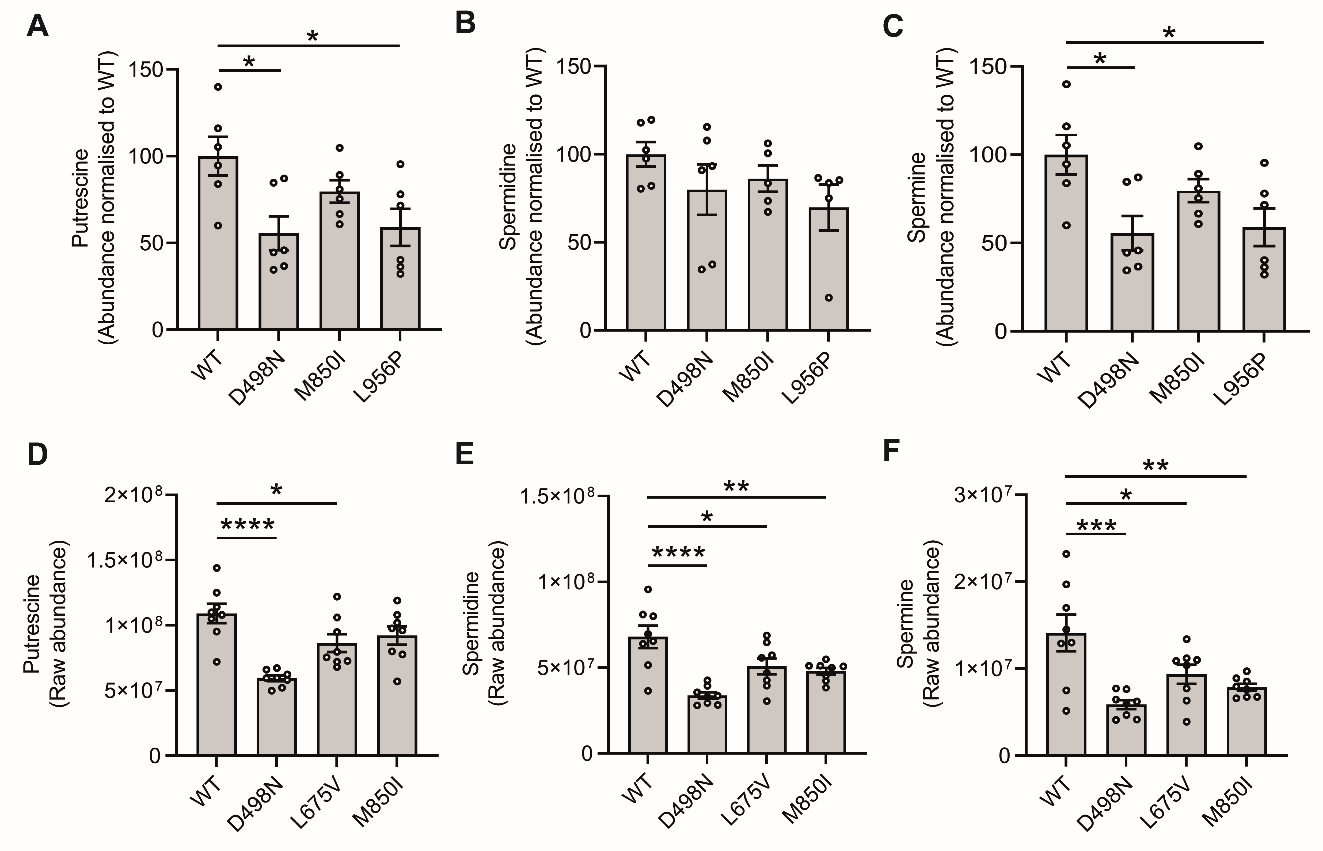
**

**Supplement Figure 8. Basal polyamine levels are not reduced in HMEC-1 stables, but are reduced in SH-SY5Y cells overexpressing the M850I ATP13A3 variant.** (A-C) Metabolomics data showing the normalised basal intracellular levels of (A) putrescine, (B) spermidine and (C) spermine in HMEC-1 cells over-expressing ATP13A3 WT (WT), D498N and the L675V and M850I PAH missense variants. (D-F) Metabolomics data showing the total basal intracellular levels of (D) putrescine, (E) spermidine, and (F) spermine in SH-SY5Y cells over-expressing ATP13A3 WT, an artificial transport dead mutant (D498N) and the L675V and M850I PAH missense variants. Mean ± SEM are shown and data were analysed by one-way ANOVA with Tukey’s post hoc test for multiple comparisons. *P<0.05, **P<0.005, ***P<0.001 and ****P<0.0001.


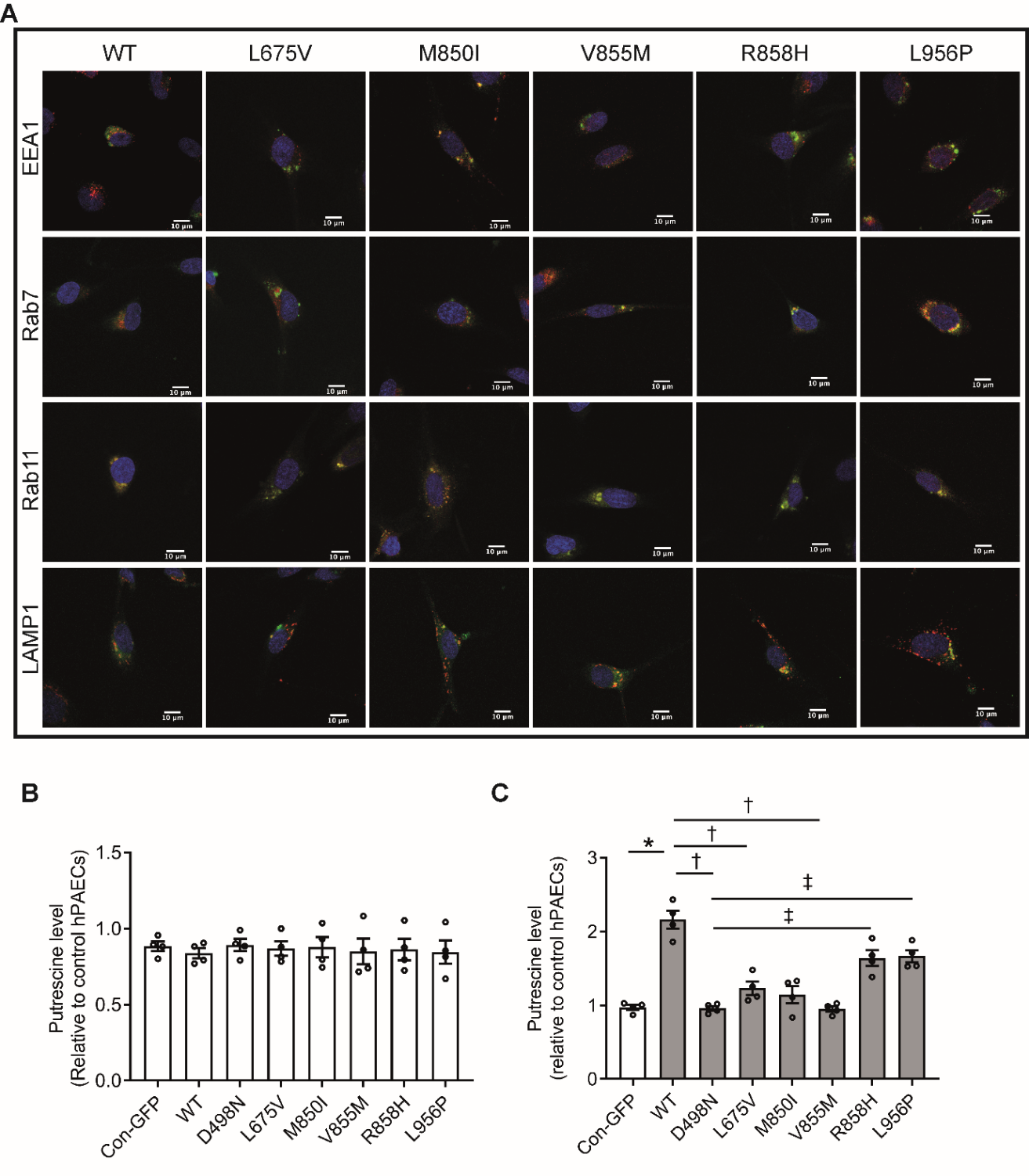


**Supplement Figure 9. PAH associated variants impair ATP13A3 mediated putrescine uptake in endothelial cells.** (A) Confocal images (63X, scale bar = 10 µm) of HMEC-1 transiently over-expressing GFP-tagged wild type (WT) or PAH-associated variants (L675V, M850I, V855M, R858H, L956V) GFP-*hATP13A3* co-stained with antibodies against either EEA1, Rab7, Rab11 or LAMP1. (B-C) Cellular putrescine in hPAECs overexpressing lentiviruses encoding ATP13A3 WT, D498N, PAH-associated variants or GFP-tagged empty vectors. Cells were incubated overnight in EBM2 containing: (B) 2%FBS alone or (C) with 1mM Putrescine. Data (n=4 experiments) are polyamine peak area ratio relative to sample protein concentration normalised to the GFP-empty vector. Data are mean ± SEM analysed by one-way ANOVA with Tukey’s post hoc test for multiple comparisons *P<0.05 compared with con-GFP. ^†^P<0.05 compared with WT, ^‡^P<0.05 compared with D498N.

**
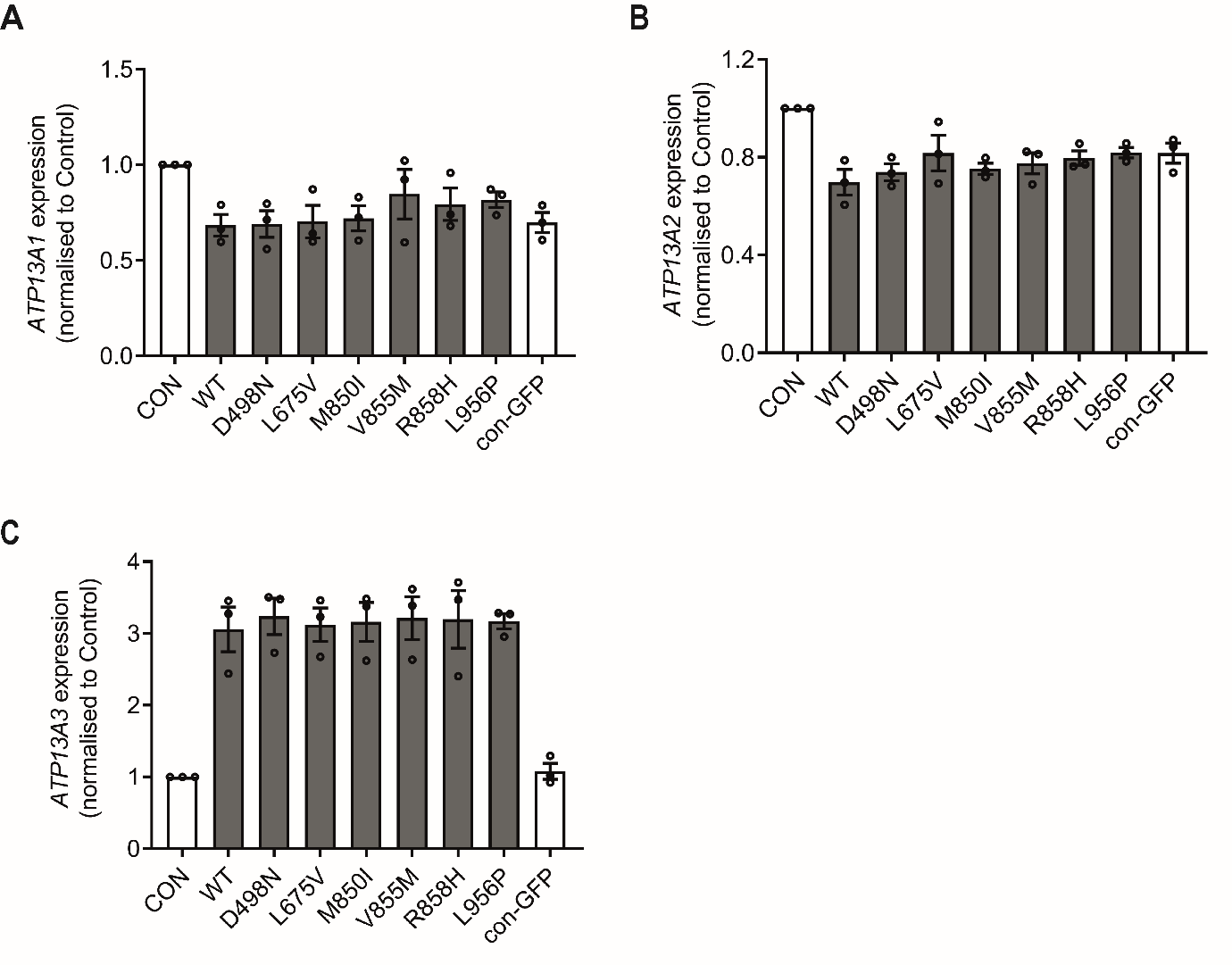
**

**Supplement Figure 10. mRNA expression of P5-type ATPases in hPAECs transiently overexpressing WT and PAH-associated ATP13A3 variants.** mRNA expression of (A) *ATP13A1* (B) *ATP13A2* (C) *ATP13A3* of control hPAECs (CON), and hPAECs transiently transduced with lentiviruses encoding GFP-tagged ATP13A3 WT (WT), an artificial transport dead mutant (D498N), PAH-associated variants (L675V, M850I, V855M, R858H, L956P) or non-targeted control (con-GFP). Data (n=3 experiments) are mean ± SEM and presented as fold-change relative to DH1.

**
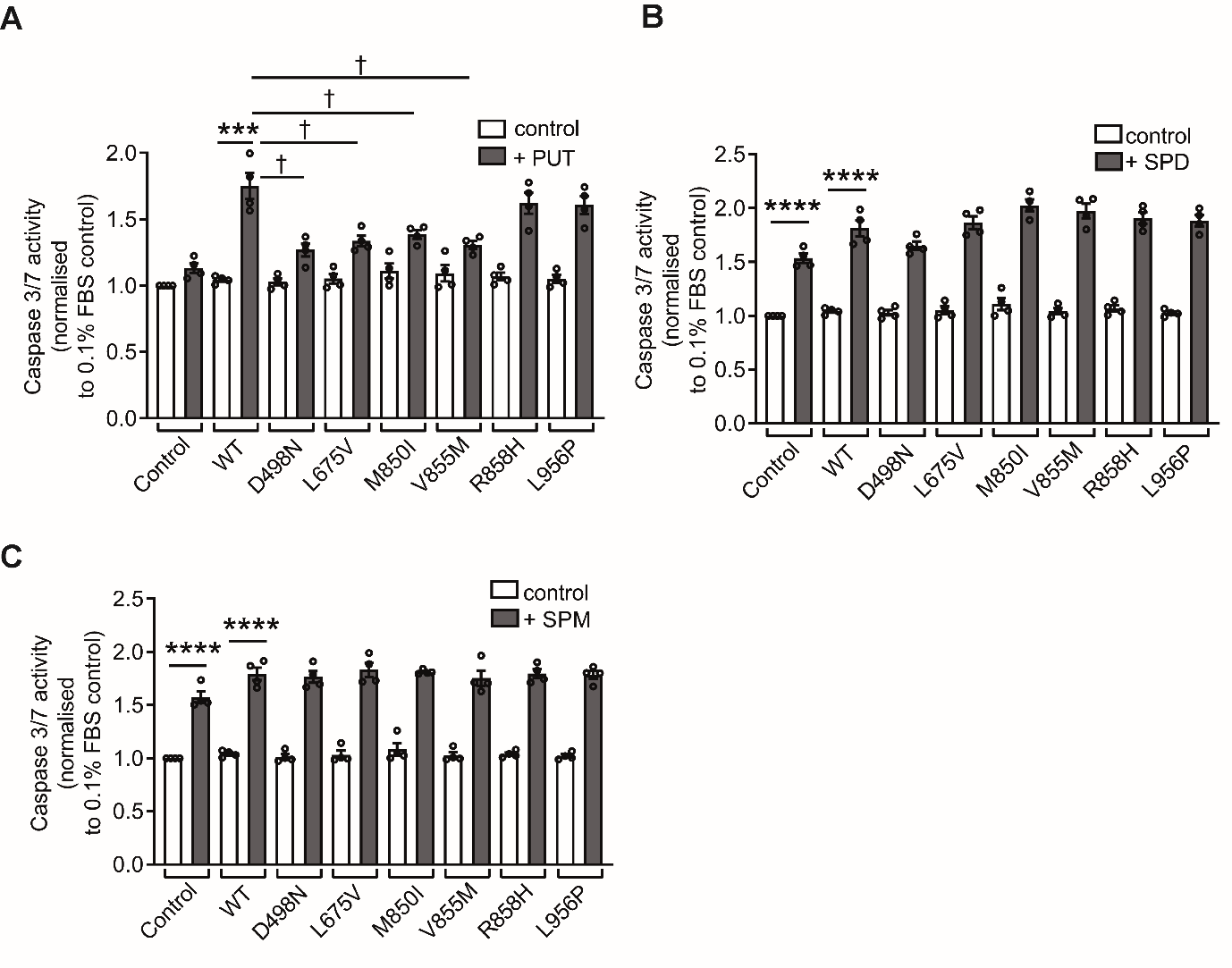
**

**Supplement Figure 11. ATP13A3 regulates the endothelial response to polyamine-induced stress.** Apoptosis of hPAECs transduced with lentiviruses encoding GFP-tagged *ATP13A3* WT, an artificial D498N mutant and PAH-associated variants (L675V, M850I, V855M, R858H, L956P)*.* Cells were cultured overnight in EBM2 containing 0.1%FBS with or without (A)10mM putrescine (PUT), (B)1mM spermidine (SPD) or (C)1mM spermine (SPM) and apoptosis assessed by Caspase-Glo®3/7 assay. Data (n=4 experiments) are mean ± SEM of the fold change relative to hPAECs cultured in 0.1% FBS. ***P<0.001, ****P<0.0001 unpaired t-test compared with cells in 0.1%FBS. ^†^P<0.05 one-way ANOVA with Dunnett's test for multiple comparisons with WT

**
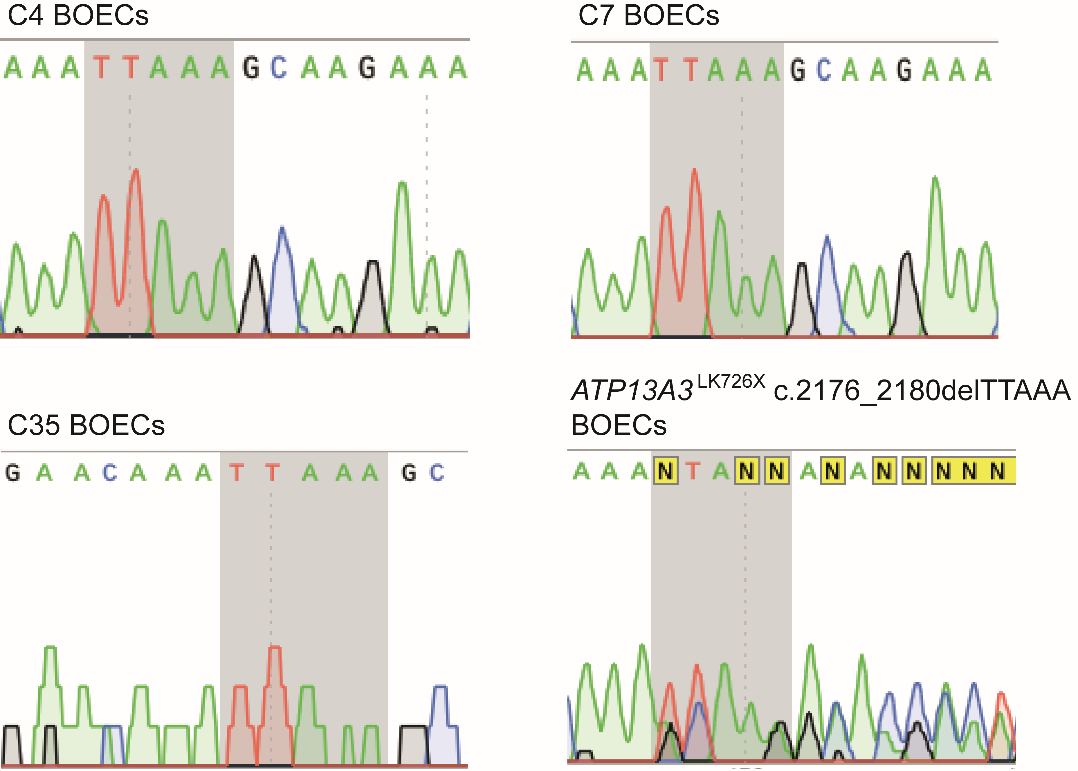
**

**Supplement Figure 12. Sanger sequencing to confirm the *ATP13A3* LK726X frameshift variant in BOECs**. Sanger sequencing data of the PCR products amplified from the genomic DNA of control BOECs (C4, C7, C35) and *ATP13A3*^LK726X^ BOECs. *ATP13A3*^LK726X^ BOECs showed the heterozygous deletion of TTAAA at the c.2176_2180 variant site.





**Supplement Figure 13. *ATP13A1*-*3* mRNA expression in control and ATP13A3^LK726X^ BOECs** mRNA expression of (A) *ATP13A3,* (B) *ATP13A1* and (C) *ATP13A2* in control BOECs (C4, C7, C35) and *ATP13A3*^LK726X^ (LK726X) BOECs. Data (n=4 experiments) are mean ± SEM presented as relative expression normalised to *B2M*. Data were analysed using a One-way ANOVA with Tukey’s post hoc test for multiple comparisons. *P<0.05, **P<0.01 .


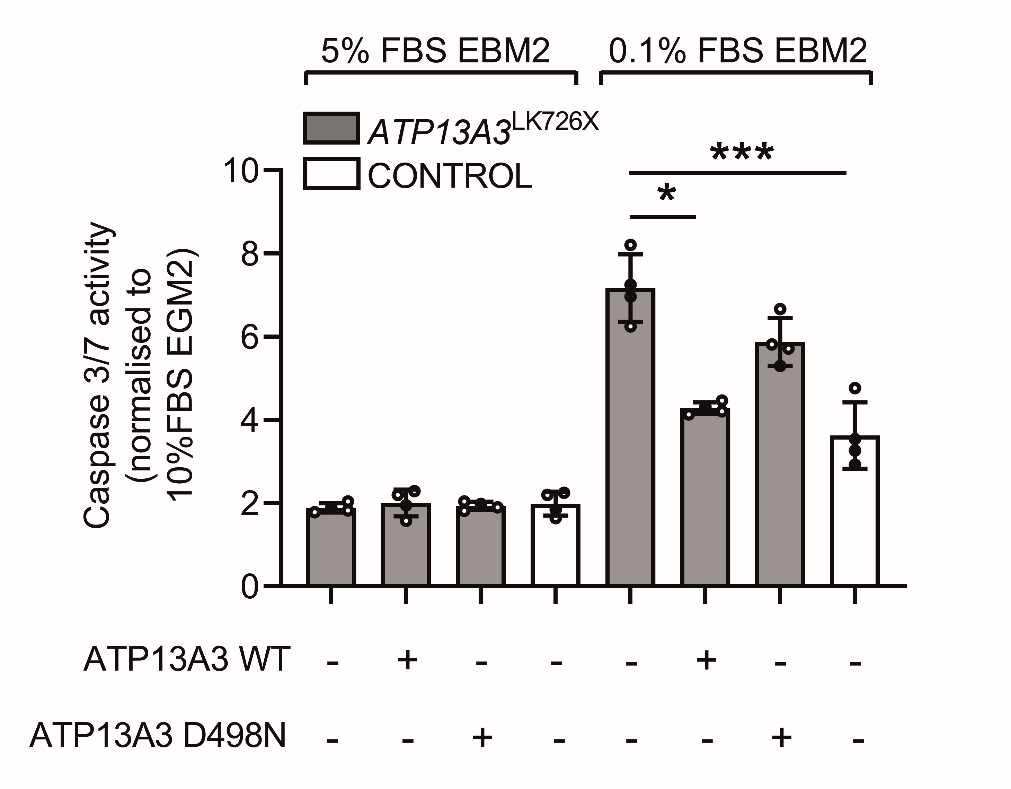


**Supplement Figure 14. Wild type *ATP13A3* overexpression rebalances the pro-apoptotic phenotype in *ATP13A3*^L726X^ BOECs.** *ATP13A3*^LK726X^ BOECs were transiently transduced with or without lentiviruses encoding ATP13A3 WT or the artificial D498N mutant. Cells cultured in in EBM2 supplemented with 5%FBS or 0.1%FBS were assessed for apoptosis by Caspase-Glo®3/7 assay. Data (n=4 experiments) are mean ± SEM analysed by one-way ANOVA with Tukey’s post hoc test for multiple comparisons *P<0.05 compared with con-GFP. ^*^P<0.05 ^***^P<0.001 compared with *ATP13A3*^LK726X^ BOECs cultured in 0.1%FBS EBM2.


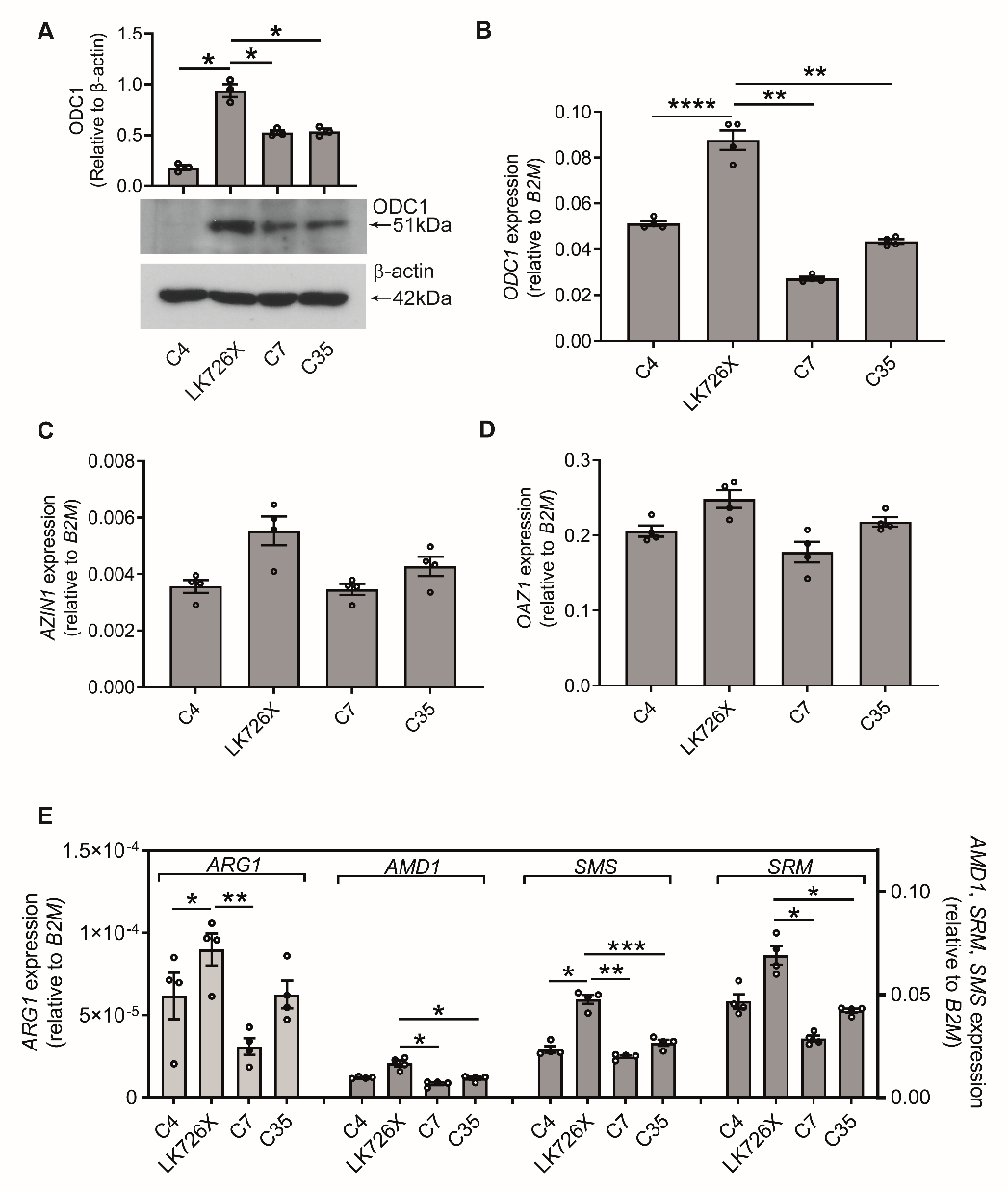


**Supplement Figure 15. The *ATP13A3* LK726X frameshift variant alters polyamine metabolism pathways.** (A) Immunoblotting of ODC1 in control BOECs (C4, C7, C35) and *ATP13A3*^LK726X^ (LK726X) BOECs. Densitometric analysis of ODC1 and β-actin was performed. (B)*ODC*, (C) *AZIN* and (D) *OAZ1* mRNA of BOECs is presented as expression relative to *B2M*. (E) mRNA expression of polyamine biosynthesis related enzymes (*ARG1, AMD1, SRM, SMS*) presented as expression relative to *B2M.* Data are representative of n=3 in (A) and n=4 in (B-E). Data (mean ± SEM) in panels A-E and were analysed using a One-way ANOVA with Tukey’s post hoc test for multiple comparisons. *P<0.05, **P<0.01, ***P<0.001 compared to *ATP13A3*^LK726X^.





**Supplement Figure 16. mRNA expression of polyamine catabolic enzymes in control and ATP13A3^LK726X^ BOECs** mRNA expression of (A) *SMOX* (B) *PAO* (C) *SAT1* in control BOECs (C4, C7, C35) and *ATP13A3*^LK726X^ (LK726X) BOECs. Data (n=4 experiments) are mean ± SEM and presented as expression relative to *B2M*.


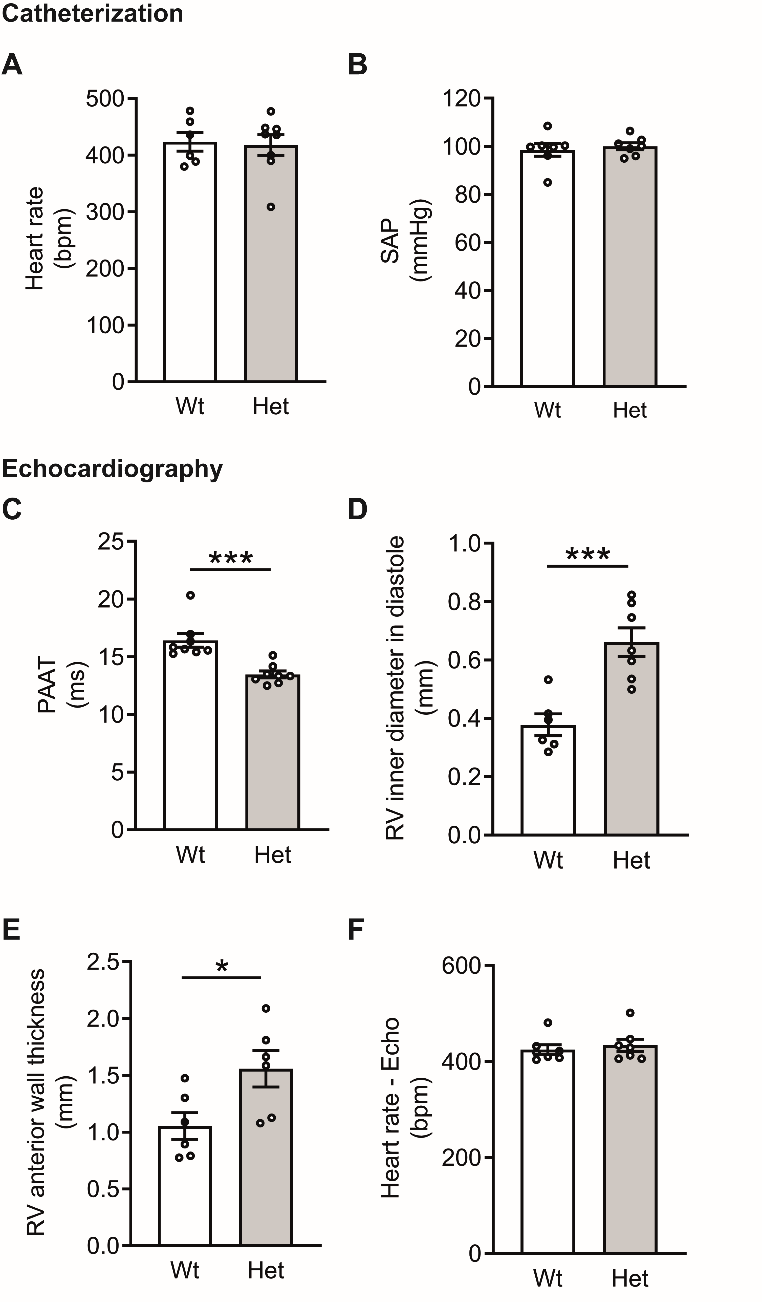


**Supplement Figure 17. Male mice harbouring an *Atp13a3*^P452Lfs^ variant spontaneously develop PAH and right ventricular dysfunction by 6 months of age.** (A-B) Invasive hemodynamic measurement of (A) heart rate and (B) systolic blood pressure (SAP) *Atp13a3*^P452Lfs^ heterozygous mice and controls (n = 6, 10). (C-F) Ultrasound echocardiography assessment of cardiopulmonary function. (C) Pulmonary artery acceleration time, (D) Right ventricular inner diameter, (E) Right ventricular end diastolic anterior wall thickness and (F) heart rate. Data are mean ± SEM analysed using an unpaired two-tailed t-test with Welch’s correction. *P<0.05, ***P<0.001

| **Cell line ID** | ***ATP13A3* variant** | **Gender** | **Age**  (years) | **Clinical details** |
| --- | --- | --- | --- | --- |
| **C1** | - | female | 45 | Healthy subject |
| **C2** | - | male | 20 | Healthy subject |
| **C4** | - | male | 30 | Healthy subject |
| **C7** | - | male | 41 | Healthy subject |
| **C26** | - | female | 25 | Healthy subject |
| **C35** | - | male | 33 | Healthy subject |
| ***ATP13A3*^LK726X^** | LK726X | male | 46 | PAH Patient  Age at diagnosis: 46 y  WHO Functional class III  mPAP: 61 mmHg  mPAWP: 22 mmHg  PVR: 9.03WU  Cardiac output (assumed fick) 4.32(L/min)  KCO 70% predicted  Exercise capacity (6MWD 30m corridor): 207 m |

**Supplement table 1. *ATP13A3* variant and demographic information of different BOEC lines**

| **HUMAN** |  |  |  |  |
| --- | --- | --- | --- | --- |
| **HGVS (protein)** | **HGVS (cDNA)** | **Exon** | **Consequence** (system) | **Variant** |
| p.Leu726AlafsTer8 | c.2176_2180  delTTAAA | 21 | Frameshift  (BOECs) | 3:194154565_CTTTAA/C |
| p.Leu675Val | c.2023T>G | 20 | Missense  (Expression) | 3:194157921_A/C |
| p.Met850Ile | c.2550G>A | 24 | Missense  (Expression) | 3:194151746_C/T |
| p.Val855Met | c.2565G>A | 24 | Missense  (Expression) | 3:194151733_C/T |
| p.Arg858His | c.2573G>A | 24 | Missense  (Expression) | 3:194151723_C/T |
| p.Leu956Pro | c.2867T>C | 27 | Missense  (Expression) | 3:194150414_A/G |
| **MOUSE** |  |  |  |  |
|  |  | **Exon** | **Consequence** | **Variant** |
| p.Pro452LeufsTer7 | c.1355delC |  | Frameshift | 6:30349828_AG/A |

**Supplement table 2. Genetic and genomic features of ATP13A3 variants assessed in this study.** Six human PAH-associated *ATP13A3* variants were studied, either as a germ-line variant in blood outgrowth endothelial cells (BOECs) or via overexpression of plasmid and lentiviral constructs (expression). The mouse strain studied harboured a homologue of a PAH-associated human ATP13A3 frameshift variant (p.Pro456LeufsTer7; c.1367delC; 3:194167785_AG/A). The human Ensembl canonical transcript used was ENST00000439040 (ATP13A3) from Genome assembly GRCh37. The mouse C57/Bl6J canonical transcript used was Genbank GCA_000001635.9 from mouse genome assembly GRCm39). HGVS:Human Genome Variation Society

| **Gene name** | **Forward 5’ - 3’** | | **Reverse 5’ - 3’** |
| --- | --- | --- | --- |
| *ATP13A1* | | AGTACGCCCATTGTGAAACT | TTGAACATCTGTAGCGTGGT |
| *ATP13A2* | | AGCTCCTCAGTTTCATCCGT | TTCCAACGGAAGAGCAGCAA |
| *ATP13A3* | | GAAAATAGGCACAGGATCAG | ATTTTACACTATGGTGGGTG |
| *ATP13A4* | | ACCCTAAGCTGGTGCCTTTC | TGGCCAGGCTGAGAAGAATG |
| *ATP13A5* | | ATTGATGGAAGCTGCGTGGT | TCTGGGGCAATGGTGTCTTT |
| *CCNA1* | ACTAGAGCAGGGGGACAGAG | | GTGTGCCGGTGTCTACTTCA |
| *CCNB1* | CTGCTGGGTGTAGGTCCTTG | | TGCCATGTTGATCTTCGCCT |
| *CCND1* | TGTCCTACTACCGCCTCACA | | CTTGGGGTCCATGTTCTGCT |
| *CCNE1* | CCACAGAGCGGTAAGAAGCA | | TAAAAGCAAACGCACGCCTC |
| *ARG1* | TGGGGAAGACACCAGAAGAA | | AGTCCGAAACAAGCCAAGGT |
| *ODC1* | ATATTGGCGGTGGCTTTCCT | | TGCAAGCGTGAAAGCTGATG |
| *AMD1* | CTAGATGTCGATGCTGGGGG | | CACAGCAAGAGTGGCAGAGA |
| *SRM* | ACCAAGTGTTACAAGCCCCA | | ACACGTGTTTGGTGAGTGAG |
| *SMS* | TGGCAGGACCATGGCTATTT | | TGAAGGTCCAGCAACACCAA |
| *SMOX* | TGGCCCTGTAGCTTTTCTTTT | | AGGCACTTACAATAACAAGGCAG |
| *PAO* | AGGGTCCTCTGGTTTTTGGT | | GCTGTGGCTGTAACTTGCTT |
| *SAT1* | TGCTGTAGATGACAACCTCCATT | | TCAAACATGCAACAACGCCA |
| *B2M* | CTCGCGCTACTCTCTCTTTCT | | CATTCTCTGCTGGATGACGTG |
| *HPRT* | GCTATAAATTCTTTGCTGACCTGCTG | | AATTACTTTTATGTCCCCTGTTGACTGG |
| *ACTB* | GCACCACACCTTCTACAATGA | | GTCATCTTCTCGCGGTTGGC |

**Supplement table 3. Primer sequences for human genes analysed using qPCR**

| Antibody | Source | Dilution | Secondary | Dilution |
| --- | --- | --- | --- | --- |
| Rabbit  anti-ATP13A3 | Sigma-Aldrich  HPA029471 | 1:200 | rabbit Alexa Fluor®488 | 1:200 |
| Mouse Anti-  VE-Cadherin CD144 | BD Biosciences  555661 | 1:200 | Mouse Alexa Fluor®568 | 1:200 |
| Mouse Anti-EEA1 | BD  Biosciences  610456 | 1:200 | Mouse Alexa Fluor®568 | 1:200 |
| Mouse Anti-Rab7 | SANTA CRUZ  sc-376362 | 1:200 | Mouse Alexa Fluor®568 | 1:200 |
| Mouse Anti-Rab11a | SANTA CRUZ  Sc-166912 | 1:200 | Mouse Alexa Fluor®568 | 1:200 |
| Mouse Anti-LAMP1 | SANTA CRUZ  Sc-20011 | 1:200 | Mouse Alexa Fluor®568 | 1:200 |

**Supplement table 4. antibodies used for cellular immunostaining.**

**Complete unedited Western blots**


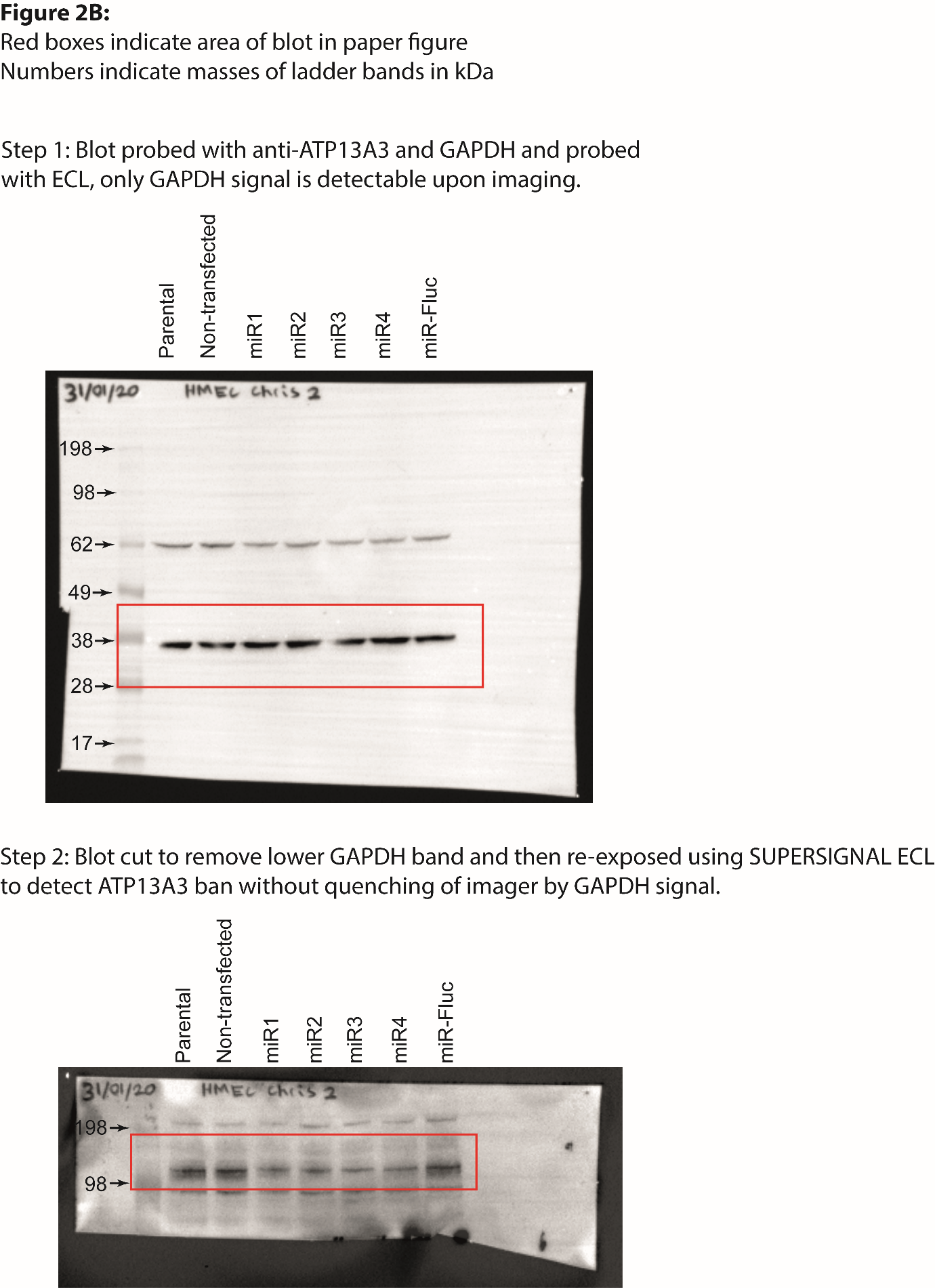


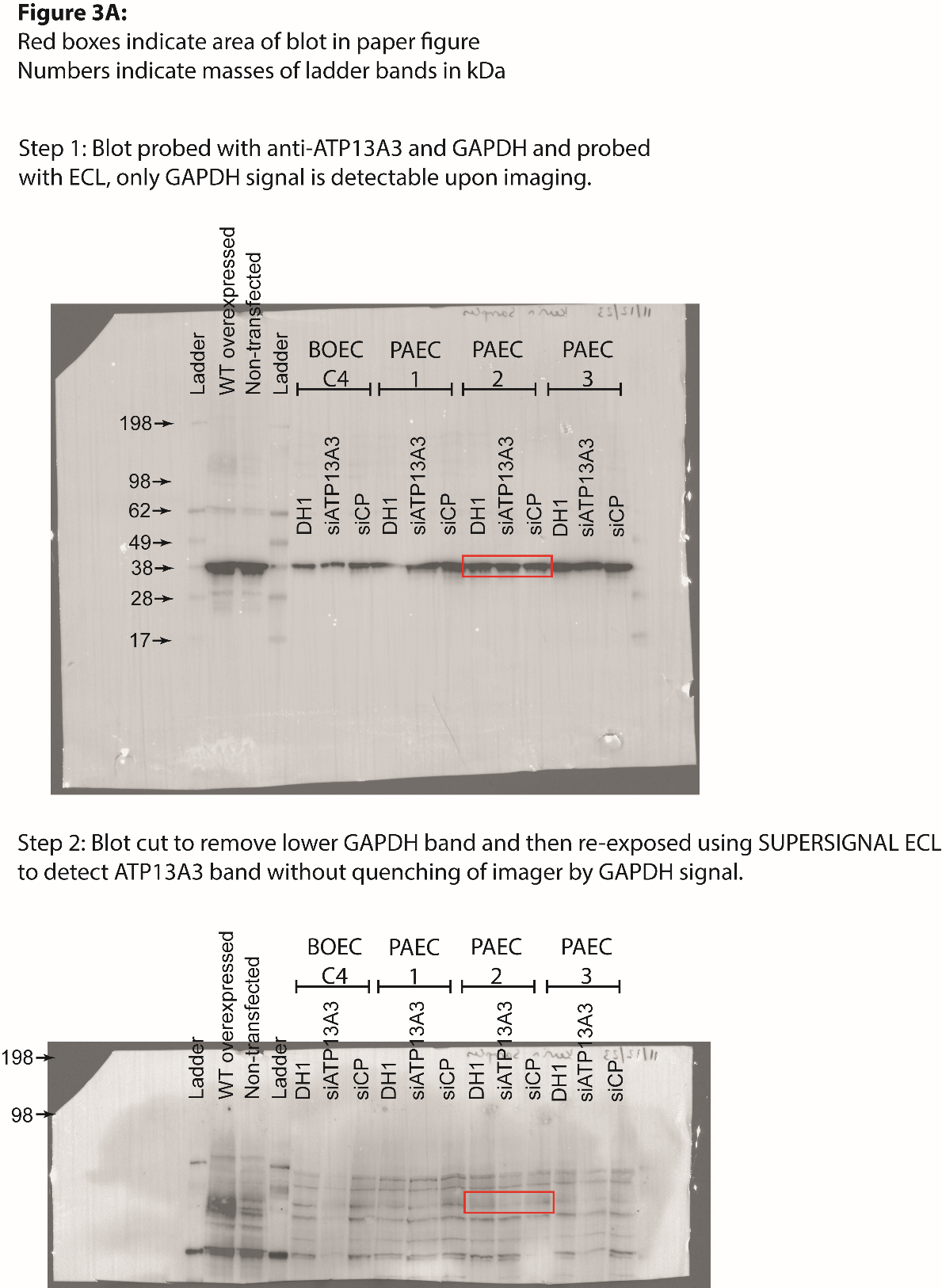


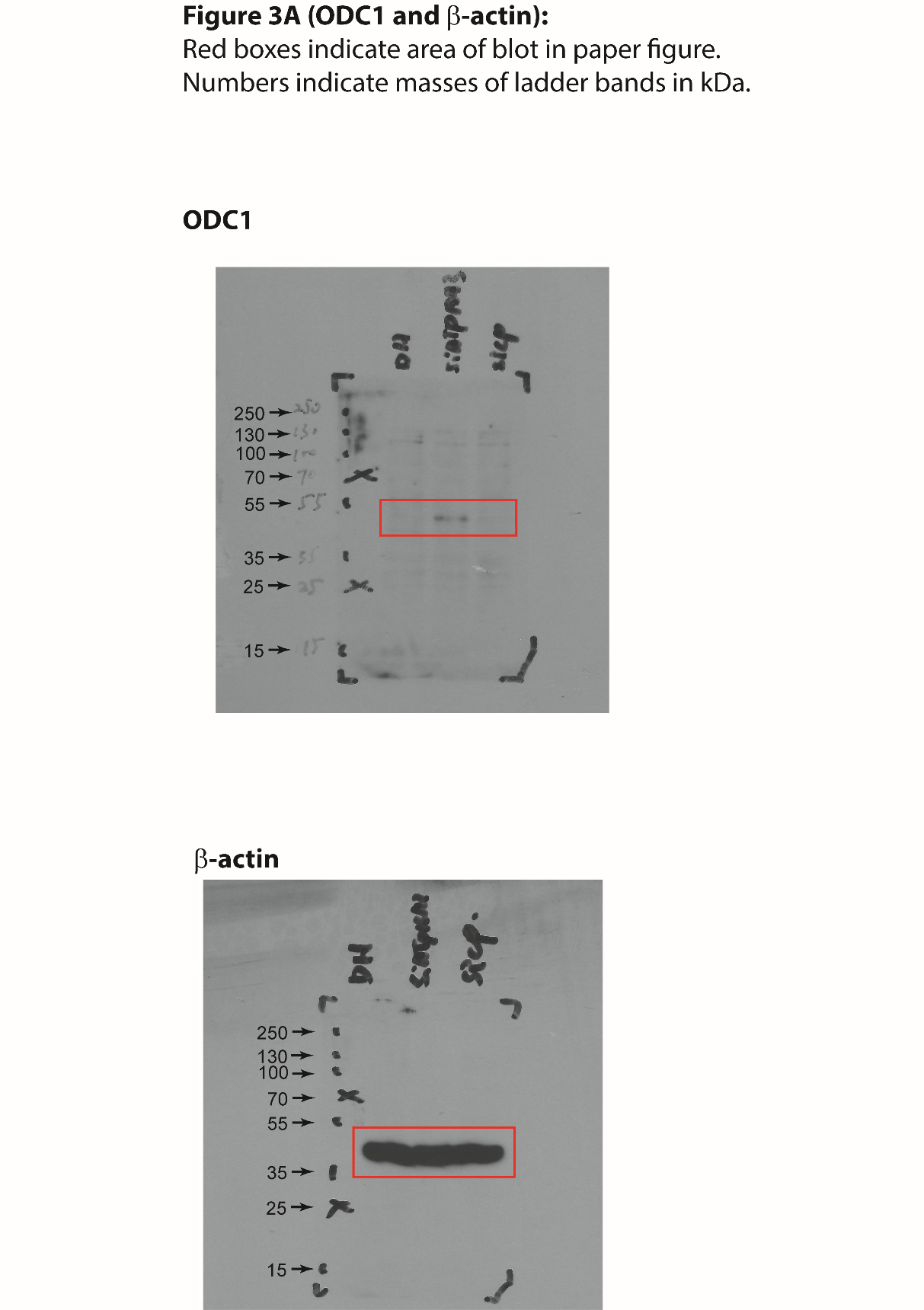


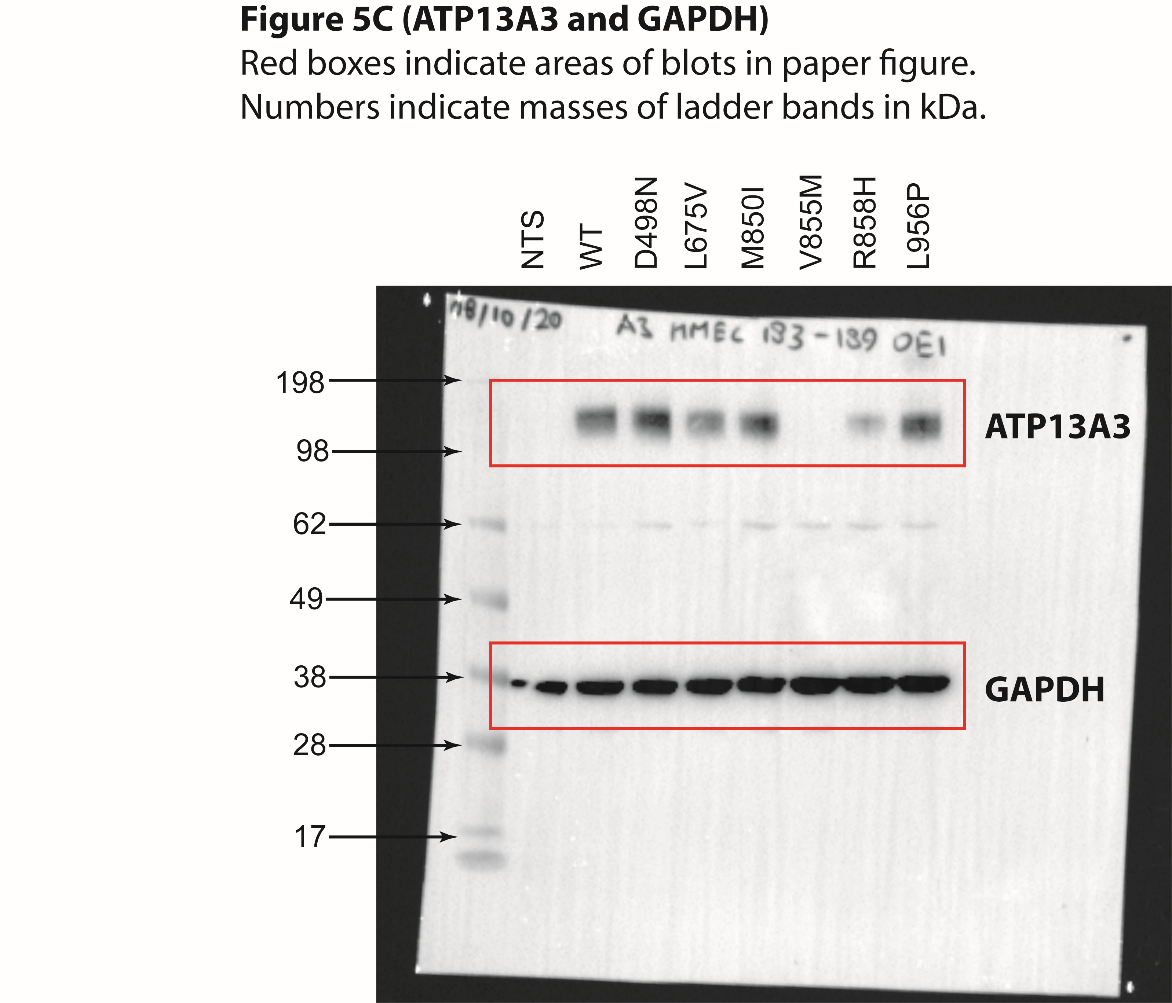


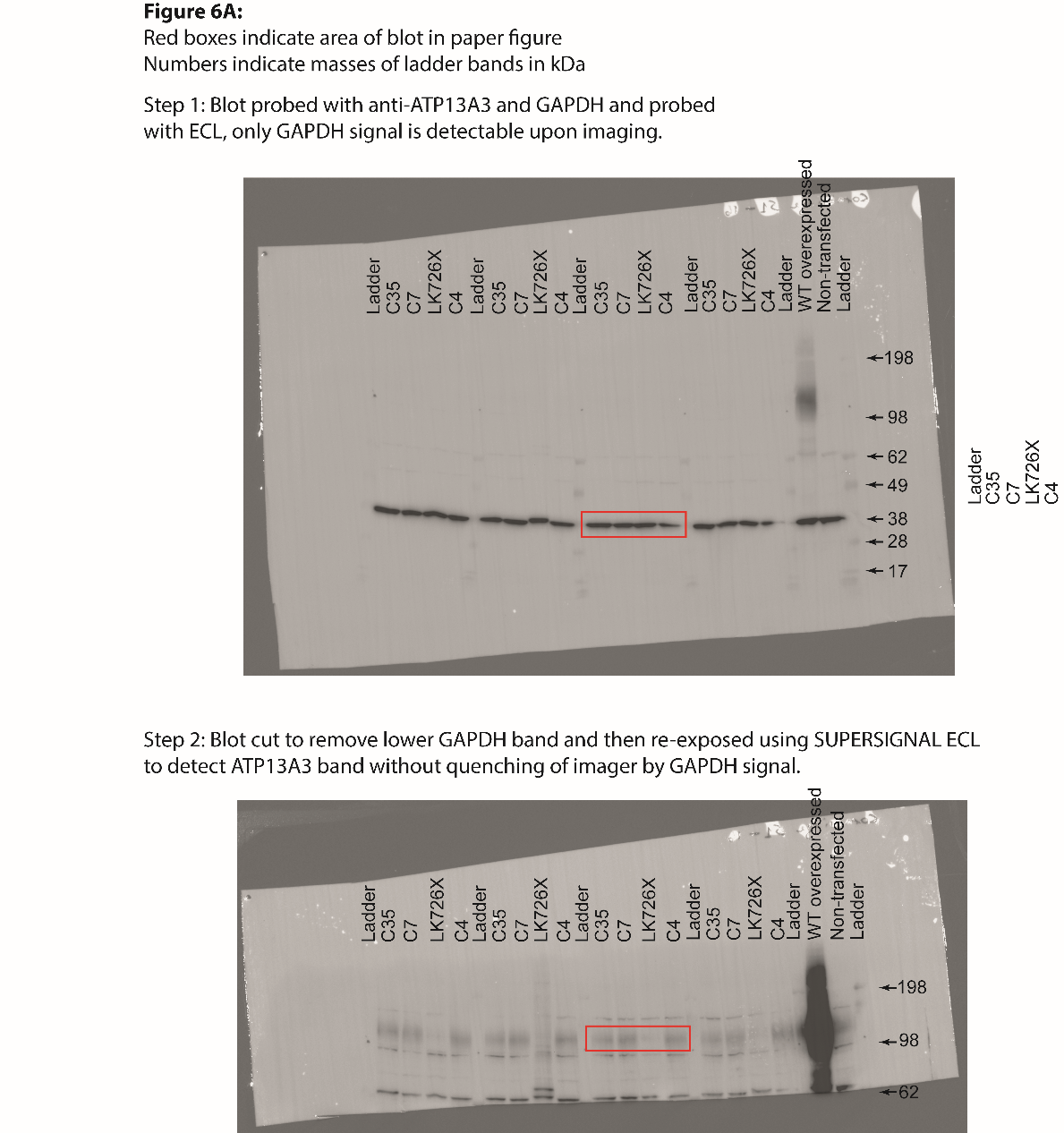


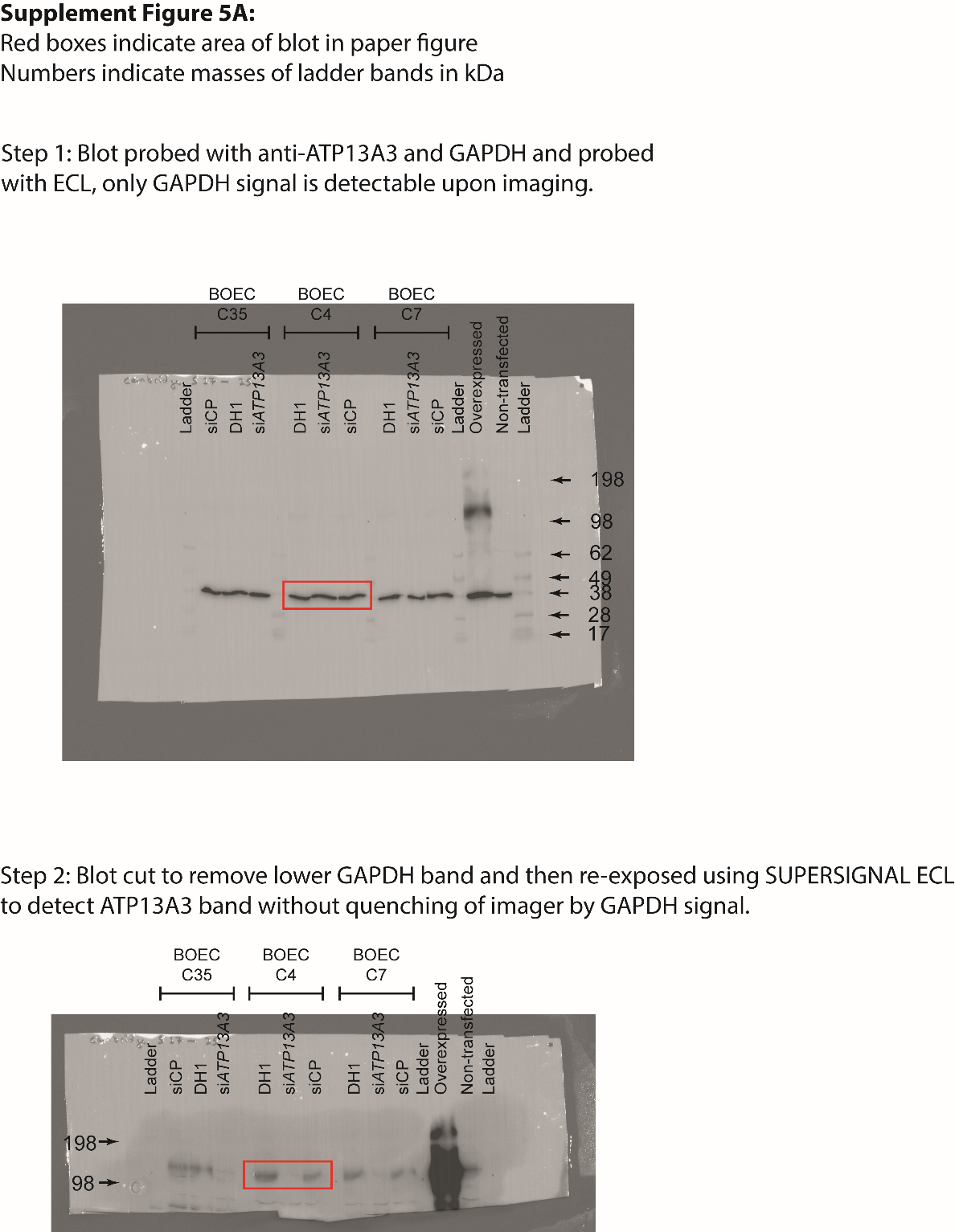


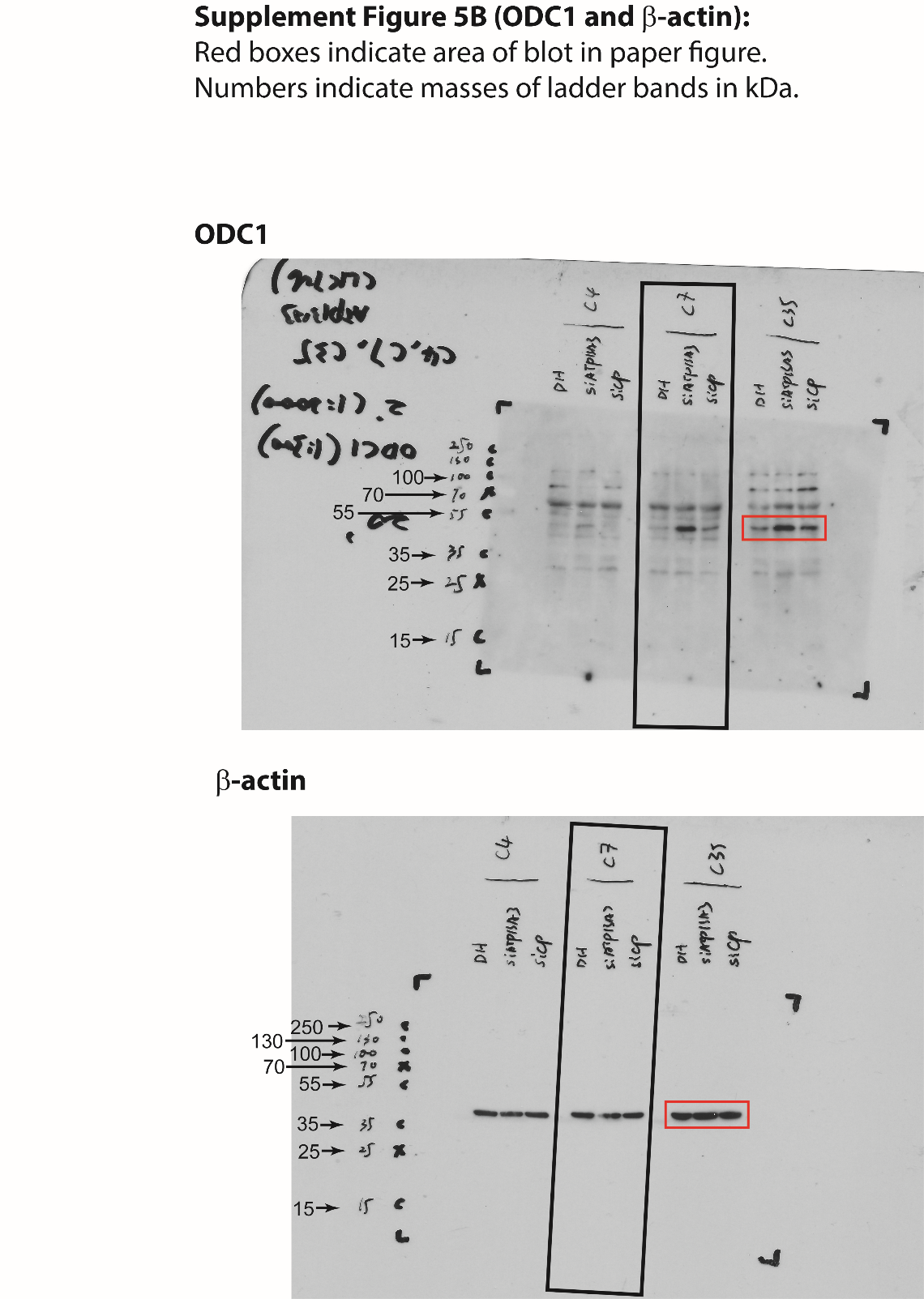


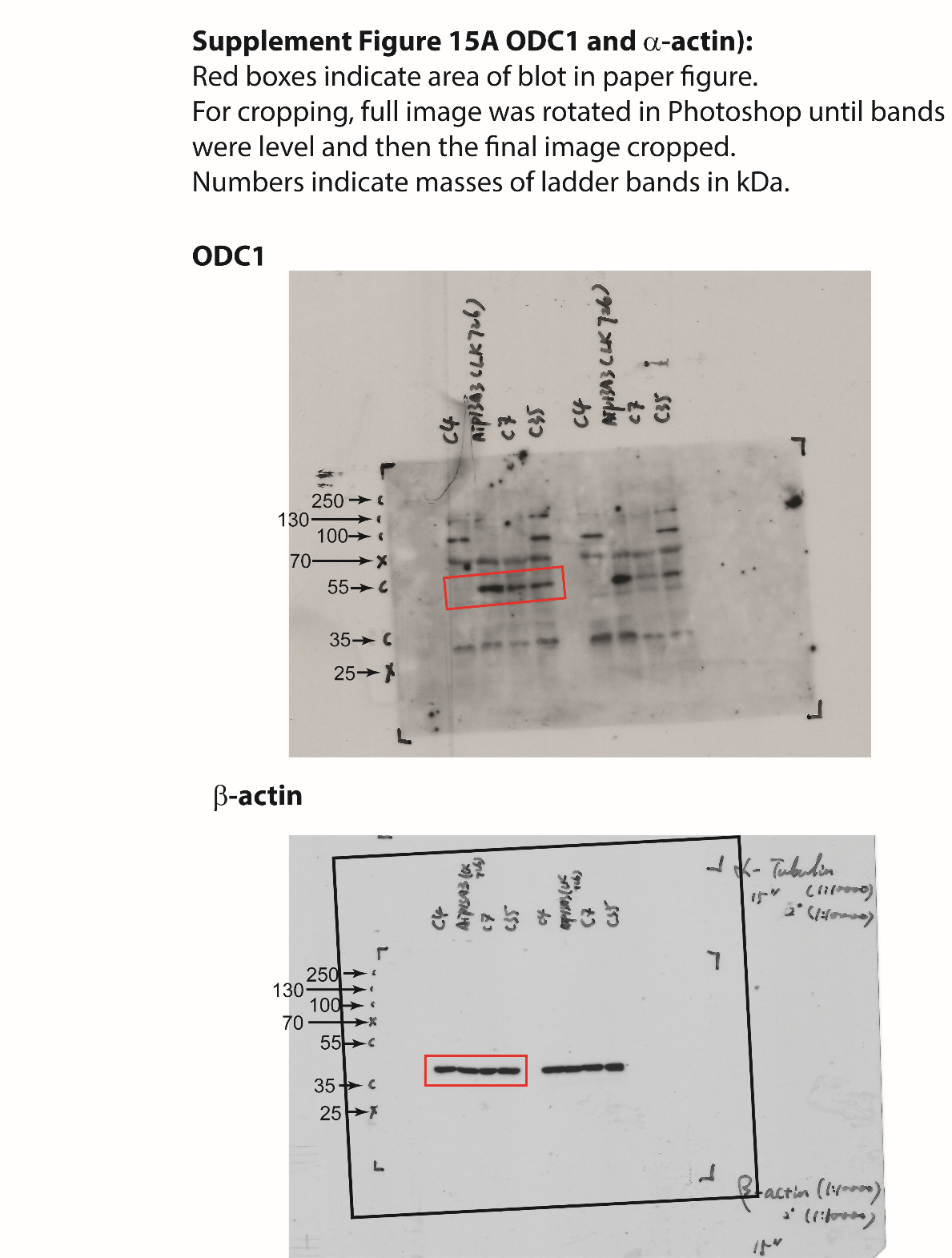

Supplement: cvae068_Supplementary_Data [file cvae068_supplementary_data.docx]
